# A widespread sequence-specific mRNA decay pathway mediated by hnRNPs A1 and A2/B1

**DOI:** 10.1101/gad.277392.116

**Published:** 2016-05-01

**Authors:** Rene Geissler, Alfred Simkin, Doreen Floss, Ravi Patel, Elizabeth A. Fogarty, Jürgen Scheller, Andrew Grimson

**Affiliations:** 1 Department of Molecular Biology and Genetics, Cornell University, Ithaca, New York 14853, USA;; 2 Institute of Biochemistry and Molecular Biology II, Medical Faculty, Heinrich-Heine-University, 40225 Düsseldorf, Germany;; 3 Graduate Field of Genetics, Genomics, and Development, Cornell University, Ithaca, New York 14853, USA

**Keywords:** post-transcriptional gene regulation, *cis*-regulatory element, 3′ UTR, mRNA decay, CCR4–NOT deadenylase complex, hnRNPs A2/B1 and A1

## Abstract

3′-untranslated regions (UTRs) specify post-transcriptional fates of mammalian messenger RNAs (mRNAs), yet knowledge of the underlying sequences and mechanisms is largely incomplete. Here, we identify two related novel 3′ UTR motifs in mammals that specify transcript degradation. These motifs are interchangeable and active only within 3′ UTRs, where they are often preferentially conserved; furthermore, they are found in hundreds of transcripts, many encoding regulatory proteins. We found that degradation occurs via mRNA deadenylation, mediated by the CCR4–NOT complex. We purified *trans* factors that recognize the motifs and identified heterogeneous nuclear ribonucleoproteins (hnRNPs) A1 and A2/B1, which are required for transcript degradation, acting in a previously unknown manner. We used RNA sequencing (RNA-seq) to confirm hnRNP A1 and A2/B1 motif-dependent roles genome-wide, profiling cells depleted of these factors singly and in combination. Interestingly, the motifs are most active within the distal portion of 3′ UTRs, suggesting that their role in gene regulation can be modulated by alternative processing, resulting in shorter 3′ UTRs.

## Abstract (precis)

Geissler et al. identified two related novel 3' UTR motifs in mammals that specify transcript degradation. Degradation occurred via mRNA deadenylation, mediated by the CCR4–NOT complex. They purified *trans* factors that recognize the motifs and identified hnRNPs A1 and A2/B1.

Regulation of gene expression is central to the understanding of biological systems; while transcription is the predominant stage of regulation, consequential regulation also acts on the other processes that together culminate in protein synthesis. The collection of post-transcriptional events that act on mature messenger RNAs (mRNAs) are governed chiefly by regulatory *cis*-acting sequences within the 5′ and 3′ untranslated regions (UTRs), which recruit *trans* factors that largely control mRNA stability, localization, and translation. In mammals, 3′ UTRs in particular direct diverse regulatory outcomes and are known to play significant roles in the regulation of a growing number of mRNAs. Identifying the sequences within 3′ UTRs that direct regulation and elucidating the underlying mechanisms are essential for a comprehensive understanding of regulatory biology.

A wide variety of post-transcriptional events is triggered by sequences within 3′ UTRs. mRNA decay and translation are frequently modulated, in particular by AU-rich elements (AREs) and microRNA (miRNA) target sites (Garneau et al. 2007; Barrett et al. 2012). In response to various cellular signals, AREs interact with different sets of RNA-binding proteins (RBPs), some of which recruit mRNA decay enzymes such as exonucleases or endonucleases to enhance deadenylation and/or degradation, whereas others increase mRNA stability or control translation. Prominent examples of ARE-binding proteins include AUF1, KSRP, TTP, and TIA-1, which interact with the exosome, the deadenylases PARN and CCR4–NOT, and translation initiation factors (Chen et al. 2001; Gherzi et al. 2004; Lykke-Andersen and Wagner 2005; Tao and Gao 2015). In opposition, HuR competes for ARE binding to increase mRNA stability (Lal et al. 2004). Target sites for miRNAs recruit a miRNA–protein complex, which binds 3′ UTRs in a sequence-specific manner and interacts with multiple decay and translation initiation factors to trigger transcript destabilization and translational repression (Bartel 2009; Braun et al. 2011; Chekulaeva et al. 2011; Fukao et al. 2014). Importantly, although diverse *cis* and *trans* factors are known to regulate 3′ UTRs, this knowledge is far from sufficient to explain the extensive variation in mRNA translation and stability across the transcriptome (Matoulkova et al. 2012).

Multiple lines of evidence establish that many additional, yet to be described regulatory pathways act on 3′ UTRs. First, comparative genomic studies of mammalian 3′ UTRs identified an abundance of significantly conserved sequence tracts within 3′ UTRs, only a modest fraction of which correspond to recognized regulatory motifs (Siepel et al. 2005; Xie et al. 2005). Second, biochemical approaches have established that a large fraction of 3′ UTR sequence is protein-bound (Baltz et al. 2012); however, the identities, functions, and specific binding sites of only a small fraction of these proteins are known (Cook et al. 2011). Third, detailed studies of individual 3′ UTRs have revealed many regulatory sites within specific 3′ UTRs and have established that the overall impact of a 3′ UTR derives from the combined actions of numerous discrete contributions (Didiano and Hobert 2008; Kristjansdottir et al. 2015). Finally, 3′ UTRs themselves are not static, with alternative cleavage and polyadenylation (APA) generating shorter and longer isoforms in different cellular environments (Mayr 2015). Taken together, these observations imply that detailed mechanistic descriptions of 3′ UTR-mediated activities are a prerequisite for understanding their roles in regulatory pathways.

Here, we identified and investigated a pair of related novel regulatory motifs, which are found in ∼7% of human 3′ UTRs. We establish that these motifs are repressive, recruiting the CCR4–NOT complex to deadenylate and destabilize host transcripts. The heterogeneous nuclear ribonucleoproteins (hnRNPs) A2/B1 and A1 recognize the motif and are both required for the underlying regulation. We examined the role of hnRNPs A2/B1 and A1 genome-wide by depleting cells for both proteins and profiling the transcriptome; these experiments established a major novel role for these proteins acting together in controlling the stability of hundreds of transcripts. Interestingly, the motifs regulate transcripts encoding subunits of the CCR4–NOT complex itself, suggesting the existence of a feedback loop. Additional features of these motifs are their significant enrichment in transcripts encoding key regulatory proteins and their tendency to function when located in the 5′ and 3′ edges of 3′ UTRs, regions that are least and most effected, respectively, by APA. Together, our results provide the first insights into and mechanistic explanation of the roles of hnRNPs A2/B1 and A1 in a novel and extensive mRNA decay pathway.

## Results

### Many novel *cis*-regulatory elements exist within mammalian 3′ UTRs

**Figure 1.**
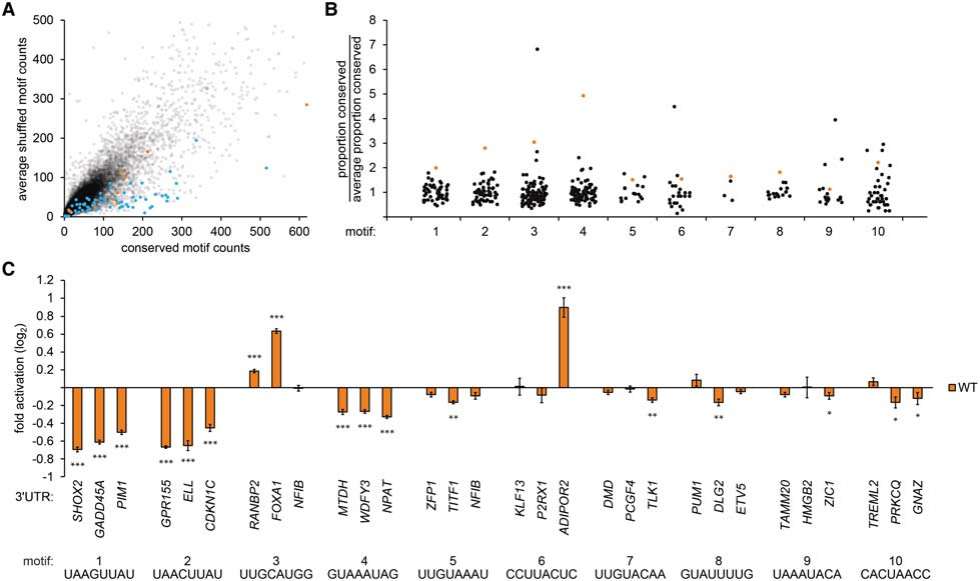
Identification of *cis*-regulatory elements in mammalian 3′ UTRs. (*A*) Count of conserved instances in human, mouse, rat, and dog 3′ UTRs for each 8-nt sequence (*X*-axis) compared with the average count of corresponding shuffled sequences (*Y*-axis). Target sites for conserved miRNAs (blue dots) and selected novel motifs (orange dots) are indicated. (*B*) Counts of conserved instances for each of 10 novel 3′ UTR sequence motifs (orange dots) compared with conserved counts of corresponding shuffled motifs (black dots); counts were normalized to the average count of each set of shuffles. (*C*) Validation of candidate regulatory elements in luciferase reporter assays. Each group of bars displays reporter activities for a 3′ UTR sequence motif assayed in three exemplar contexts. Luciferase activity of constructs with intact motifs (orange bars) was normalized to otherwise identical constructs with mutated elements. Error bars indicate standard errors. *n* = 9. (***) *P* < 0.001; (**) *P* < 0.01; (*) *P* < 0.05, Wilcoxon rank-sum test.

To identify novel regulatory elements within 3′ UTRs, we used a comparative approach to systematically search 3′ UTRs across the human genome, searching for motifs with evidence of selection in four mammalian genomes. For each 8-nucleotide (nt) sequence, we calculated the number of conserved instances of the 8mer within mammalian 3′ UTRs together with an estimate of the number of conserved instances that we would expect if the 8mer were evolving without selection. This approach was based on a proven strategy developed to examine miRNA-binding sites within 3′ UTRs (Lewis et al. 2005). Most sequences exhibited little evidence of selection; however, motifs corresponding to miRNA-binding sites tended to show strong evidence of selection (Fig. 1A, blue points), as expected (Lewis et al. 2005). Consistent with previous studies (Siepel et al. 2005; Xie et al. 2005), many motifs that do not correspond to miRNA-binding sites or other known elements also exhibited strong evidence of selection.

To investigate whether conserved sequence motifs within 3′ UTRs confer regulatory functions, we tested 10 candidate motifs (Fig. 1B) for their regulatory potential in tissue culture reporter assays. We selected only motifs without detectable sequence overlap with any human miRNA seed matches (Kozomara and Griffiths-Jones 2013), the polyadenylation signal AAUAAA (Beaudoing et al. 2000), pumilio-binding sites UGUAHAUA (Li et al. 2010), and the ARE consensus sequence UUAUUUAWW (Lagnado et al. 1994). Due to the impact of local sequence context, not every instance of an element may be functional. Therefore, we selected three different exemplar 3′ UTRs for each motif. To assess their functionality, ∼500-nt fragments of each 3′ UTR, centered on a motif, were cloned into a luciferase reporter. As a control, we generated paired reporters in which three nucleotides within a motif were mutated; mutations were selected so that the resulting sequences did not exhibit any unusual patterns of conservation. Notably, all 10 motifs exhibited significant modulation of reporter expression in at least one 3′ UTR (Fig. 1C), indicating that each of these motifs corresponds to *cis*-regulatory elements. Although most elements were repressive, several were activating. Two of the elements, themselves similar in sequence (UAAGUUAU and UAACUUAU), mediated strong repression in all six different sequence contexts that we tested.

### The 3′ UTR element UAASUUAU is broadly repressive and prevalent

**Figure 2.**
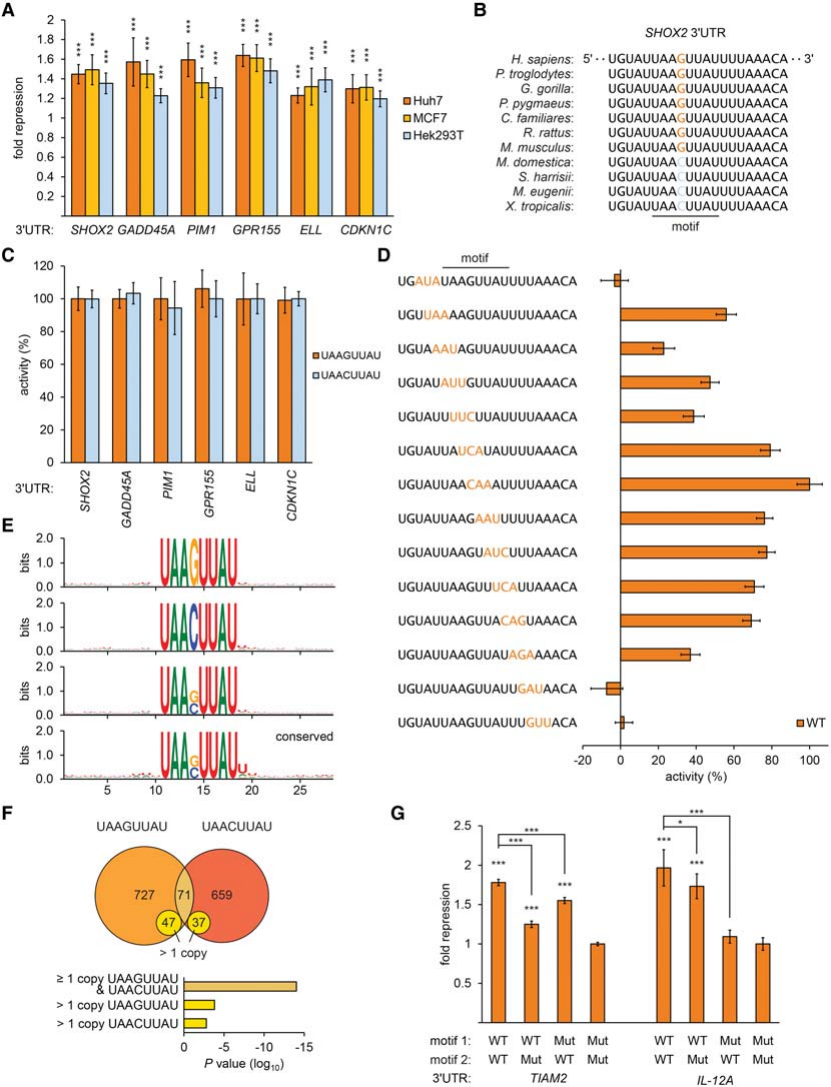
Characterization of a prevalent 3′ UTR regulatory element. (*A*) Motif-mediated repression across a range of cell types. Reporter assays of exemplar 3′ UTRs containing the UAASUUAU motif, normalized to otherwise identical mutated reporters, plotting fold repression; otherwise as described in Figure 1C. (*B*) Sequence alignment of the motif in *SHOX2* 3′ UTRs. (*C*) Reporter assays exchanging UAAGUUAU and UAACUUAU; otherwise as described in *A*. (*D*) Mutational analysis of the motif in *SHOX2* 3′ UTRs. Luciferase reporter assays of a series of constructs containing sequence changes (indicated in orange) flanking and within the motif. Fold repression was calculated for reporters containing the wild-type sequence and each mutant; repressive activity of each mutant, relative to wild type, is shown; otherwise as described in *A*. (*E*) Web logos of 3′ UTR sequences centered on all instances of the motif (*top* three panels for UAAGUUAU, UAACUUAU, and UAASUUAU) and all conserved instances (*bottom* panel for UAASUUAU). (*F*, *top*) Venn diagram illustrating the number and overlap of 3′ UTRs containing the motif. (*Bottom*) Hypergeometric tests indicating that more 3′ UTRs contain multiple copies of the motif than expected if co-occurrence were random. (*G*) Multiple copies of the motif are functional in 3′ UTRs. Luciferase activities of constructs containing two copies of the motif were compared with otherwise identical constructs with either or both elements mutated; otherwise as described in *A*.

We focused exclusively on further characterization of the related sequences UAAGUUAU and UAACUUAU (UAASUUAU) because of their presence and conservation in many 3′ UTRs and because our preliminary reporter experiments (Fig. 1C) demonstrated that individual examples typically mediate regulation of a magnitude at least equal to established elements such as miRNA-binding sites (Grimson et al. 2007). Both elements were functional in diverse cellular environments, as judged by reporter assays performed in different cell lines (Fig. 2A), suggesting that the corresponding *trans* factors are expressed broadly. Because the two elements differ by only a single nucleotide and occur alternatively in orthologous 3′ UTRs (Fig. 2B; Supplemental Fig. S1A), we suspected that they share a common mechanism. In support of this idea, sequence changes that convert between the elements have no effect, indicating that either element can substitute fully for the other (Fig. 2C).

It was formally possible that the functional element corresponded to the mutated sequence that we selected as a control. To address this possibility, we created concatemerized reporter constructs in which a short region of the homeobox family member *SHOX2* and the G-protein-coupled receptor *GPR155* 3′ UTRs containing the motif were repeated (Supplemental Fig. S1B). As we increased the number of copies of the wild-type element, we observed additional repression. In contrast, additional copies of the mutated element had a negligible effect (Supplemental Fig. S1C–F). Together, these results confirm that the sequence UAASUUAU is repressive.

To assess whether the sequence UAASUUAU per se results in the regulation that we observed, we performed a mutational analysis across the element and surrounding nucleotides within the *SHOX2* 3′ UTR. Mutations disrupting any of the 8 nt that comprise the element impaired or inactivated the regulatory impact of the element, with mutations in the center of the 8mer tolerated least (Fig. 2D). In contrast, reporters containing mutations in nucleotides flanking the 8mer had little or no effect on the function of the element. To corroborate this conclusion, we examined the sequence preferences surrounding the motif (Fig. 2E), finding little evidence for additional sequence preferences, and established that the motif was sufficient to mediate repression when introduced into 3′ UTRs (Supplemental Fig. S1G). Together, these data suggest that the sequence UAASUUAU itself and alone corresponds to the regulatory element.

The sequence UAASUUAU is found within the 3′ UTRs of 1315 human mRNAs, with 142 3′ UTRs (∼11%) harboring multiple copies of the element (median spacing between elements of ∼780 nt), representing a significant enrichment of 3′ UTRs containing multiple copies (Fig. 2F). To test whether additional copies of the element potentiate repression in a native context, we examined 3′ UTRs containing multiple copies. Reporter constructs based on the guanine nucleotide exchange factor *TIAM2* or cytokine subunit of *interleukin-12A* 3′ UTRs, each of which contains a pair of elements, revealed that the degree of repression increases in response to additional copies (Fig. 2G). Together, these results identify the sequence UAASUUAU (referred to here as the motif) as a novel 3′ UTR regulatory element that is both broadly active and found in a large fraction of transcripts.

### The motif is preferentially found in genes encoding regulatory proteins

**Figure 3.**
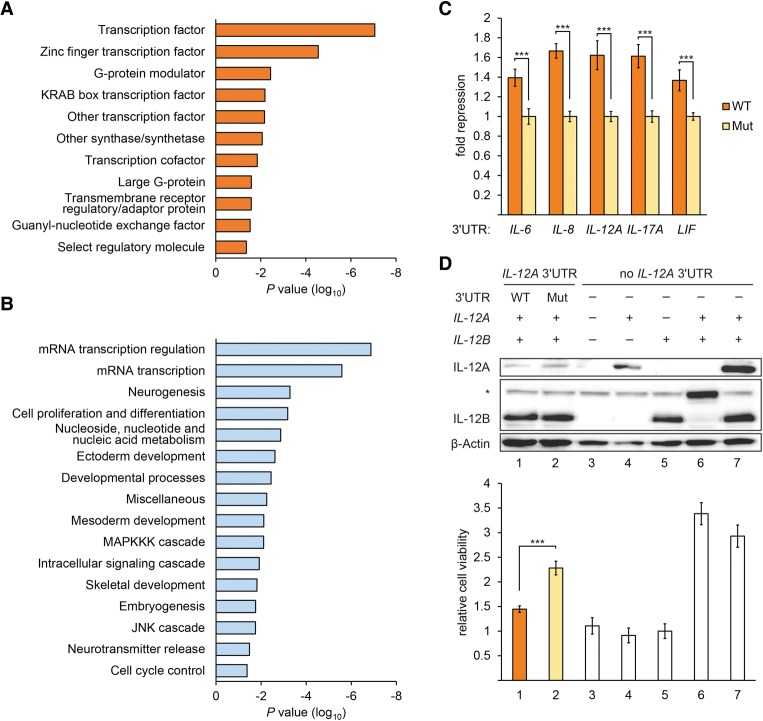
The motif is enriched in 3′ UTRs of transcripts encoding regulatory proteins. (*A*,*B*) Gene ontology analysis of mRNAs whose 3′ UTRs contain the motif, reporting significantly enriched terms (*P* < 0.05) within molecular function (*A*) and biological process (*B*) categories. (*C*) Reporter assays indicating functional instances of the motif within interleukin gene 3′ UTRs; otherwise as described in Figure 1C. (*D*) IL-12 cell viability assay. (*Top* panel) Western blot probed for IL-12A, IL-12B (the asterisk denotes an unspecific band recognized by IL-12B antibody), and β-actin using protein extracts from COS-7 cells transfected with the indicated combinations of constructs expressing *IL-12B*, *IL-12A* with and without native 3′ UTR, and an *IL-12A-12B* fusion construct (lane *6*; comigrates with unspecific band). (*Bottom* panel) Cell viability assay using Ba/F3 cells expressing IL-12 receptors. Ba/F3 cells were treated with supernatants of COS-7 cells transfected with the constructs described above, and cell viability was normalized to lane *5*. *n* = 5. (***) *P* < 0.001, Student's *t*-test. Error bars denote standard deviation.

Consequential 3′ UTR regulatory elements are often enriched within transcripts encoding regulatory proteins. Gene ontology analysis revealed an enrichment of the motif within 3′ UTRs of mRNAs encoding transcription factors and regulators of proliferation and differentiation (Fig. 3A,B). To evaluate the biological significance of one class of genes enriched in the motif, we tested the efficacy and function of its regulation in interleukin signaling. Many interleukin transcripts (*interleukin-6* [*IL-6*], *IL-8*, *IL-12A*, *IL-17A*, and *LIF*) harbor the motif within their 3′ UTRs, which we confirmed were repressive (Fig. 3C). We investigated the regulatory impact of the motif in interleukin signaling using an IL-12 cell viability assay. Signaling with IL-12 occurs via cell surface receptors (IL-12Rβ1 and IL-12Rβ2), and subsequent activation and induction of transcription factors lead to cellular responses such as cell proliferation (Teng et al. 2015). IL-12 is a heterodimer encoded by *IL-12B* and *IL-12A* which contains two copies of the motif. The secreted heterodimer can activate IL-12 receptors (Teng et al. 2015). IL-12 was expressed in COS-7 cells by cotransfecting cDNAs encoding *IL-12B* and *IL-12A* with either the wild-type 3′ UTR or a version in which the motif was mutated (Fig. 3D, top). An *IL-12B-IL-12A* fusion construct was transfected as a control (Fig. 3D, top, lane 6). *IL-12A* expression of constructs with the wild-type motif was decreased, as expected. After treatment of Ba/F3 cells, which express IL-12 receptors, with cell culture supernatants from transfected COS-7 cells, viability of Ba/F3 cells was assayed (see the Materials and Methods). Cell viability was significantly reduced in response to supernatants from cells transfected with the native *IL-12A* construct compared with supernatants from cells expressing *IL-12A* not subject to regulation by the motif (Fig. 3D, bottom). Together, these data indicate that regulation by the motif is sufficient to modify the biological outcome of IL-12 signaling. Given the high frequency of the motif in 3′ UTRs and its propensity to be active, we reasoned that the underlying regulation might be important in a variety of biological contexts; here, we concentrated on establishing the underlying mechanism.

### Post-transcriptional regulation of transcripts containing the motif

**Figure 4.**
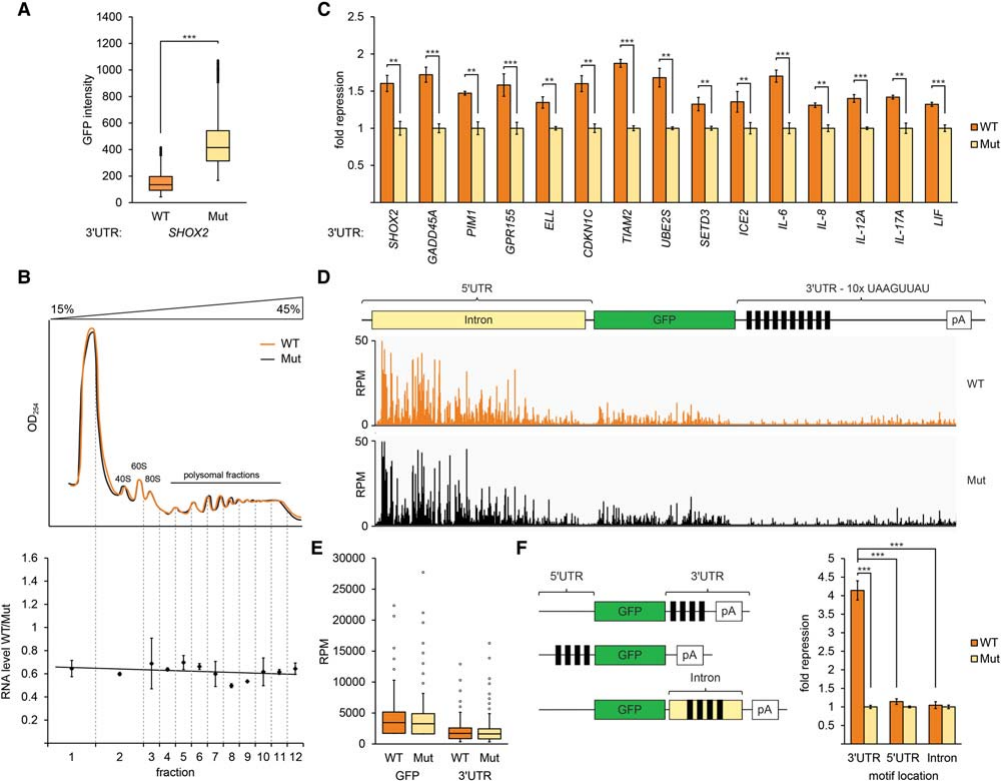
The 3′ UTR motif UAASUUAU destabilizes host mRNAs. (*A*) Box plot of GFP intensities from A549 cells containing stably integrated *GFP* reporters with the wild-type *SHOX2* 3′ UTR or with the motif mutated (Mut) (*P* < 0.001). (*B*) RNA steady-state levels, and not translation, are regulated by the motif. (*Top*) Polysome gradient fractionation of cells stably expressing wild-type (orange) and Mut (black) *GFP* reporters described in *A*. RNA quantification of wild-type compared with Mut reporters (*Y*-axis) assessed by quantitative RT–PCR (qRT–PCR) on each fraction (*X*-axis). *n* = 2. Error bars denote standard deviation. (*C*) The motifs are functional within diverse 3′ UTRs. RNA levels, assessed by qRT–PCR, from stably integrated *GFP* constructs containing the indicated 3′ UTRs, compared between 3′ UTRs containing intact and mutated copies of the motif. *n* = 3. (***) *P* < 0.001; (**) *P* < 0.01, determined with Student's *t*-test. Error bars denote standard deviation. (*D*) The motif does not regulate transcription. PRO-seq (precise nuclear run-on and sequencing) analysis of *GFP* constructs containing 10 copies of the wild-type motif or mutated (Mut) copies showing reads per million mapped reads (*Y*-axis) values across the transcript (*X*-axis) together with a transcript schematic. (*E*) Box plot of reads per million expression values from *D* mapped to the coding sequence and 3′ UTR downstream from the motif. (*F*) Activity of the motif is restricted to the 3′ UTR. Illustration of reporter constructs; black rectangles denote the motif. qRT–PCR of RNA levels from the illustrated constructs. *n* = 3. (***) *P* < 0.001, determined with Student's *t*-test. Error bars indicate standard deviation.

To investigate the molecular mechanism of the repressive function mediated by the motif, we established cell lines stably expressing *GFP* 3′ UTR reporter constructs. In addition to facilitating biochemical investigations, integrated reporters better recapitulate endogenous gene expression than do transiently transfected reporter constructs (Smith and Hager 1997). Flow cytometry analysis of GFP expressed from *GFP-SHOX2* 3′ UTR constructs revealed significantly reduced GFP levels when compared with otherwise identical reporters in which the motif was mutated (Fig. 4A), confirming our results derived from transiently transfected luciferase reporters.

To assess whether translational repression reduced reporter protein levels, we analyzed the *GFP-SHOX2* 3′ UTR mRNAs in polysome profiles (Fig. 4B). If the motif represses translation, we would expect wild-type mRNAs to be translated by fewer ribosomes than otherwise identical mRNAs with mutated copies of the motif and, consequently, a shift of the mRNA population from heavy to light polysomes. Analysis of reporter transcripts, however, indicated that mRNAs with wild-type or mutated motifs sedimented equivalently. Importantly, wild-type mRNA reporter levels were reduced to ∼60% of the levels of the mutant reporter in all fractions (Fig. 4B). To confirm that the motif controls mRNA levels, we extended our analyses of integrated *GFP* reporters to additional examples of 3′ UTRs harboring it; in all cases, we observed reductions in steady-state mRNA levels that were dependent on the presence of the motif (Fig. 4C).

To examine whether motif-mediated reductions in mRNA levels derive from transcriptional or post-transcriptional events, we conducted a PRO-seq (precise nuclear run-on and sequencing) experiment using cells expressing *GFP* 3′ UTR constructs containing 10 copies of the motif (or mutated control sequences) derived from the *SHOX2* 3′ UTR. The resulting map of actively transcribing RNA polymerase II demonstrated that transcription is unaffected by the presence of the motif (Fig. 4D,E). Furthermore, we compared the activity of the motif in reporter constructs in which the motif was located in the 3′ UTR, 5′ UTR, or an intron. Importantly, motif activity was exclusive to a location within the 3′ UTR (Fig. 4F; Supplemental Fig. S2). Taken together, these results indicate that the motif acts post-transcriptionally to modulate mRNA levels.

### 3′ UTRs containing the UAASUUAU element trigger CCR4–NOT complex-mediated deadenylation

**Figure 5.**
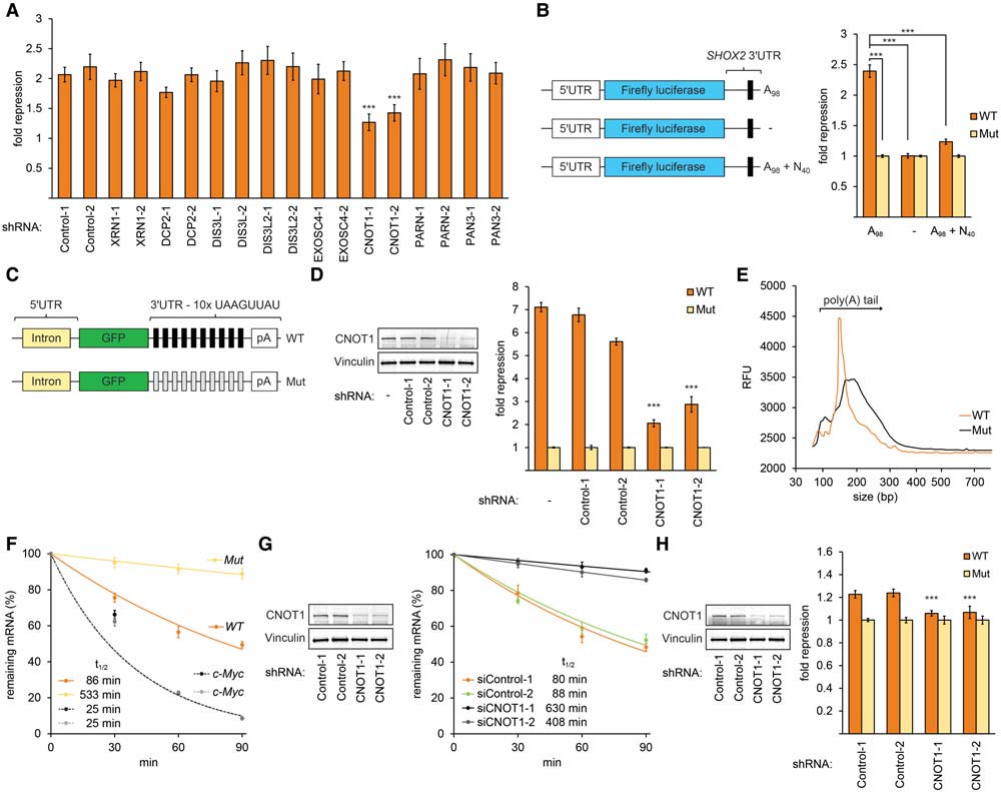
The motif promotes deadenylation by recruitment of the CCR4–NOT complex. (*A*) CNOT1 is required for motif activity. Luciferase assays performed on transfected RNAs containing the *SHOX2* 3′ UTR with a poly(A)_98_ tail performed in A549 cells depleted for the indicated mRNA decay factors. *n* = 9. (***) *P* < 0.001, Wilcoxon rank-sum test. Error bars denote standard deviation. (*B*) A functional poly(A) tail is necessary for motif activity. (*Left*) Illustration of luciferase reporter mRNAs; black rectangles denote the motif, A_98_ denotes a poly(A) tail of 98 adenine nucleotides, and N_40_ denotes a tail of 40 non-A nucleotides. (*Right*) Firefly luciferase activities of the illustrated constructs (orange) compared with activities of otherwise identical RNAs containing a mutation disrupting the motif (yellow). Reporters were transfected into A549 cells, and activities were normalized to cotransfected Renilla luciferase mRNAs. *n* = 9. (***) *P* < 0.001, Wilcoxon rank-sum test. Error bars denote standard deviation. (*C*) Schematic of *GFP* constructs containing 10 copies of wild-type (black rectangles) and mutated (gray rectangles) motifs. (*D*, *right* panel) qRT–PCR analysis of *GFP* RNA levels expressed from constructs presented in *C* in CNOT1- and control-depleted A549 cells. *n* = 6. (***) *P* < 0.001, Student's *t*-test. Error bars display standard deviations. (*Left* panel) Validation of shRNA knockdown efficacy. Western blots probed with antibodies recognizing CNOT1 and vinculin using CNOT1-depleted extracts and control extracts; CNOT1 protein levels were reduced to 17%–26%, respectively. Vinculin was probed as a loading control. (*E*) Poly(A) tail length analysis of *GFP* reporters described in *C*. Poly(A) tails were RT–PCR-amplified and assessed for size (*X*-axis) and intensity (*Y*-axis, relative fluorescence intensity) using a fragment analyzer. (*F*) RNA decay analysis of mRNAs expressed from *GFP* constructs shown in *C*. Decay kinetics of reporter transcripts analyzed by qRT–PCR after treatment with actinomycin D (orange and yellow lines). Dashed lines indicate endogenous *c-myc* RNA decay in cell lines expressing the wild-type and mutated *GFP* reporters (black and gray dashed lines, respectively), indicating that *c-myc* stability is comparable between reporter cell lines. Error bars represent standard errors. *n* = 6. Data were fit using exponential regression; inferred transcript half-lives are indicated. (*G*, *right* panel) RNA decay experiment as described in *F* with *GFP* constructs expressing the wild-type motif in CNOT1- and control-depleted cells. (*Left* panel) Validation of shRNA knockdown efficacy as described in *D*. (*H*) Autoregulation of CNOT1. (*Right* panel) Luciferase reporter activities of mRNAs containing the *CNOT1* 3′ UTR, which carries two copies of the motif, in CNOT1 depleted cells. Luciferase experiments were performed as described in *B*. (*Left* panel) Validation of shRNA knockdown efficacy as described in *D*.

Decay of mRNAs is mediated by multiple mechanisms, including deadenylation, decapping, and the action of 5′ and 3′ exonucleases and endonucleases (Garneau et al. 2007). To identify the decay pathway triggered by the motif, we used RNAi to deplete enzymes (Supplemental Fig. S3A,B) whose activities are required by different decay pathways and transfected in vitro transcribed, polyadenylated *SHOX2* RNA reporters with and without an intact motif. Knockdown of the deadenylase CCR4–NOT complex component CNOT1 using either of two independent shRNAs significantly impaired repressive activity of the motif. However, inhibition of the deadenylases PAN2/PAN3 or PARN or other RNA decay enzymes did not have a significant effect on the activity of the element (Fig. 5A).

To examine whether the presence of the motif in a 3′ UTR promotes deadenylation, we tested in vitro transcribed luciferase reporter mRNAs containing the *SHOX2* 3′ UTR with and without an intact copy of the motif together with a poly(A)_98_ 3′ tail (Fig. 5B). As expected, when transfected into A549 cells, we observed an approximately twofold decrease in luciferase activity in the reporter containing an intact motif (Fig. 5B). We next modified this same pair of RNAs by either removing the poly(A) tail or adding an additional 40 terminal non-A nucleotides. When transfected, neither modified RNA exhibited differences in luciferase activities between RNAs with and without an intact motif (Fig. 5B). Thus, the activity of the motif requires the presence of a poly(A) tail, a conclusion that we corroborated using additional reporters (Supplemental Fig. S3C).

To further investigate the role of CCR4–NOT-mediated deadenylation in response to the motif, we generated an integrated *GFP* reporter containing multiple copies of the motif, which we reasoned would be regulated strongly by the motif, and an otherwise identical control reporter without the motif (Fig. 5C). As expected, we observed potent repression of our reporter, which was abrogated when we depleted CNOT1 (Fig. 5D). We next asked whether the motif destabilizes mRNAs through shortening of the poly(A) tail by measuring tail lengths of the same reporters. We observed a dramatic shortening of the poly(A) tail of the wild-type reporter as compared with the reporter containing mutated copies of the motif (Fig. 5E). Upon measuring decay kinetics of our reporters, we found that the wild-type reporter mRNA was destabilized compared with the control reporter (Fig. 5F). Importantly, the destabilizing effect observed for the wild-type reporter was eliminated in CNOT1-depleted cells (Fig. 5G; Supplemental Fig. S3D) and recovered by ectopic expression of shRNA-resistant CNOT1 in depleted cells (Supplemental Fig. S3E). Consequently, we conclude that the motif recruits the CCR4–NOT complex to mRNAs to promote deadenylation and destabilization.

Deadenylated mRNAs are substrates for complete degradation, typically by either 5′ or 3′ exonucleolytic decay mediated by XRN1 and the exosome, respectively (Garneau et al. 2007). We examined RNA levels of reporters with and without the motif (Fig. 5C) in XRN1- and EXOSC4-depleted cells, which revealed that RNAs with the motif are degraded further via the 5′ exonuclease XRN1 (Supplemental Fig. S3F,G).

Interestingly, the *CNOT1* 3′ UTR contains two copies of the motif, suggesting the existence of an autoregulatory feedback loop. Moreover, we confirmed that the motif is repressive in the *CNOT1* 3′ UTR (Fig. 5H). As expected, depletion of CNOT1 revealed a significant reduction in repression mediated by the motif within the *CNOT1* 3′ UTR (Fig. 5H), indicating that activity of the CCR4–NOT complex regulates the *CNOT1* transcript, potentially autoregulating global deadenylation.

### hnRNPs A1 and A2/B1 are required for repression mediated by UAASUUAU

**Figure 6.**
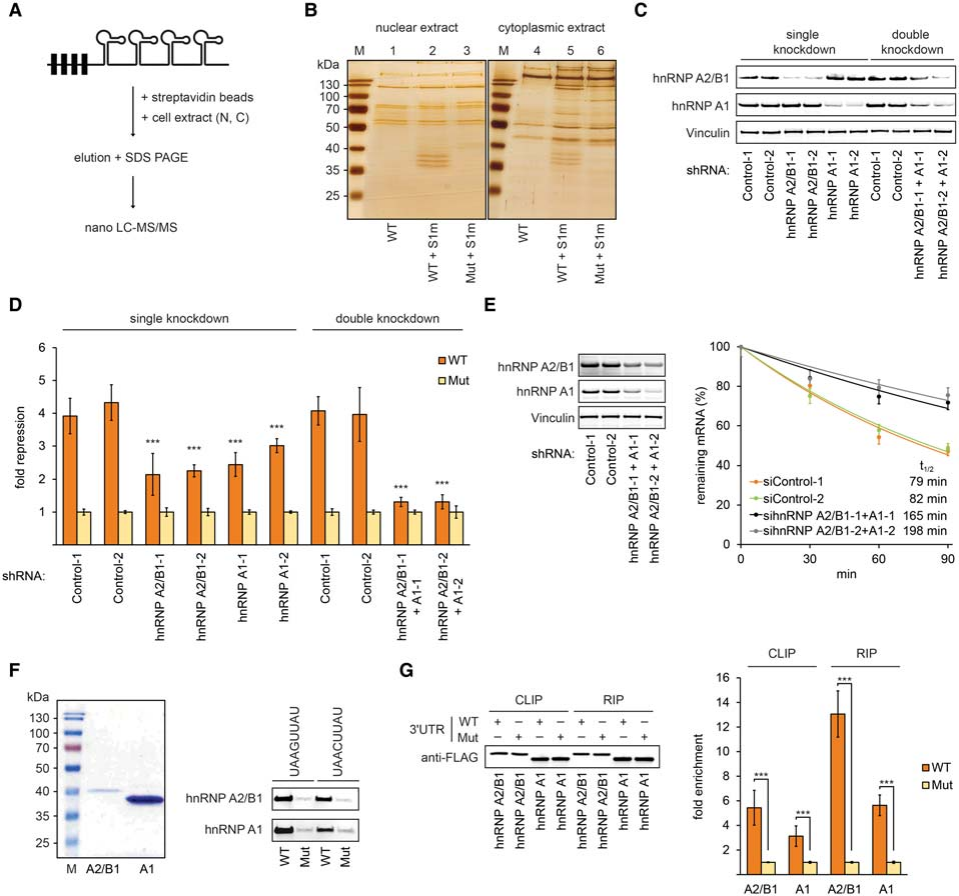
hnRNPs A2/B1 and A1 are *trans*-acting factors required for motif-mediated repression. (*A*) Schematic of pull-down experiments. RNAs were in vitro transcribed from constructs containing four copies of the motif derived from 48 nt of the *SHOX2* 3′ UTR and four copies of the S1m aptamer. After incubation with cell extracts, eluted proteins were identified by MS. (*B*) Pull-down experiments with constructs and the strategy described in *A*. Eluted proteins were separated by SDS-PAGE and silver-stained. (*C*) Validation of shRNA knockdown efficacy. Western blots probed with indicated antibodies using hnRNP A2/B1- and A1-depleted extracts; vinculin was probed as a loading control. (*D*) Luciferase assays of reporters containing four copies of the motif in hnRNP A2/B1- and A1-depleted A549 cells, as shown in *C*. *n* = 9. (***) *P* < 0.001, Wilcoxon rank-sum test. Error bars denote standard deviations. (*E*, *left* panel) Validation of shRNA knockdown efficacy as described in *C*; hnRNP A2/B1 and A1 protein levels were reduced to 33%–37% and 15%–24% in single-knockdown cells and 26% and 10%–15% in double-knockdown cells, respectively. (*Right* panel) RNA decay analysis of wild-type mRNAs expressed from *GFP* 3′ UTR constructs described in Figure 5C in hnRNP A2/B1+A1-depleted cells; otherwise as described in Figure 5F. (*F*, *left* panel) Purification of recombinant hnRNPs A2/B1 and A1 from HEK293T cells. Purified proteins were separated by SDS-PAGE and Coomassie-stained. (*Right* panel) Pull-down experiments with recombinant hnRNPs A2/B1 and A1 with constructs as described in *A*. (*G*, *left* panel) Western blot analysis of immunoprecipitated hnRNPs A2/B1 and A1 using CLIP (cross-linking and immunoprecipitation) and RIP (RNA immunoprecipitation) approaches. (*Right* panel) qRT–PCR analysis of immunoprecipitated *GFP* RNA levels (using reporters described in Fig. 5C) from HEK293T cells expressing Flag-tagged versions of hnRNPs A2/B1 and A1. *n* = 3. (***) *P* < 0.001, Student's *t*-test. Error bars display standard deviations.

To our knowledge, the CCR4–NOT complex does not possess sequence-specific binding. Instead, recruitment of CCR4–NOT to specific mRNAs is mediated by sequence-specific RNA-binding proteins (Collart 2016), such as pumilio, Nanos, TTP, or Roquin (Lykke-Andersen and Wagner 2005; Van Etten et al. 2012; Leppek et al. 2013; Bhandari et al. 2014). To identify *trans*-acting factors that recognize the sequence UAASUUAU and potentially recruit CCR4–NOT, we purified and identified proteins that bind the sequence. Our strategy (Fig. 6A) used a chimeric RNA containing multiple copies of the S1m aptamer (Leppek and Stoecklin 2014), which binds streptavidin, together with four copies of either the motif or a mutated control sequence. Three protein bands of ∼34–38 kDa were enriched from both nuclear and cytoplasmic extracts, and the enrichment of all three required the presence of both the aptamer and the motif (Fig. 6B). Using cytoplasmic extracts alone, we observed two additional protein bands of ∼68 and ∼110 kDa (Fig. 6B, lanes 4–6), which also required both the aptamer and the motif. To identify the proteins, all five enriched bands were excised and subjected to nano-liquid chromatography-tandem mass spectrometry (nano-LC-MS/MS) analysis, which identified hnRNPs A1, A2/B1, Q, and U together with additional proteins present at lower stoichiometries (Supplemental Table S1).

To validate *trans*-acting factors identified by MS, we performed luciferase reporter assays with A549 cells stably expressing shRNAs corresponding to each potential factor (Fig. 6C; Supplemental Fig. S4A). Reporters contained four copies of the motif or mutated controls, and two independent shRNAs were used for each knockdown experiment. Depletion of hnRNP Q or hnRNP U, which were found in the 68- and 110-kDa bands, respectively, did not affect luciferase activity of the wild-type reporters, nor did depletion of additional candidates tested (Supplemental Fig. S4B). In contrast, knockdown of either hnRNP A2/B1 or A1 significantly, albeit partially, impaired the repressive effect of the motif. Strikingly, simultaneous depletion of both A2/B1 and A1 resulted in almost complete abrogation of the repressive effect (Fig. 6D), which was recovered by ectopic expression of shRNA-resistant A1 in cells depleted of A2/B1 and A1 (Supplemental Fig. S4C). Comparable results were observed using a *SHOX2* 3′ UTR mRNA reporter (Supplemental Fig. S4D). To confirm this result, we determined the half-life of reporters containing 10 copies of the motif following depletion of both hnRNP A2/B1 and A1, which revealed significant stabilization of the mRNA compared with control-treated cells (Fig. 6E). To confirm physical interactions between A2/B1 and A1 with the motif, we repeated our pull-down strategy with purified recombinant A2/B1 and A1 and monitored the proteins by Western blotting. RNAs containing either the UAAGUUAU or UAACUUAU motif copurify with hnRNPs A2/B1 and A1; however, when the elements were mutated, neither RNA stably interacted with either protein (Fig. 6F; Supplemental Fig. S4E). This stable interaction was confirmed in vivo using both CLIP (cross-linking and immunoprecipitation) and RIP (RNA immunoprecipitation) assays, where we observed preferential binding of hnRNPs A2/B1 and A1 to RNAs containing the motif (Fig. 6G). Taken together, these results indicate that the proteins A2/B1 and A1 bind the motif and that their binding triggers increased decay of transcripts harboring the element.

### Determining the global impact of the motif on the transcriptome

**Figure 7.**
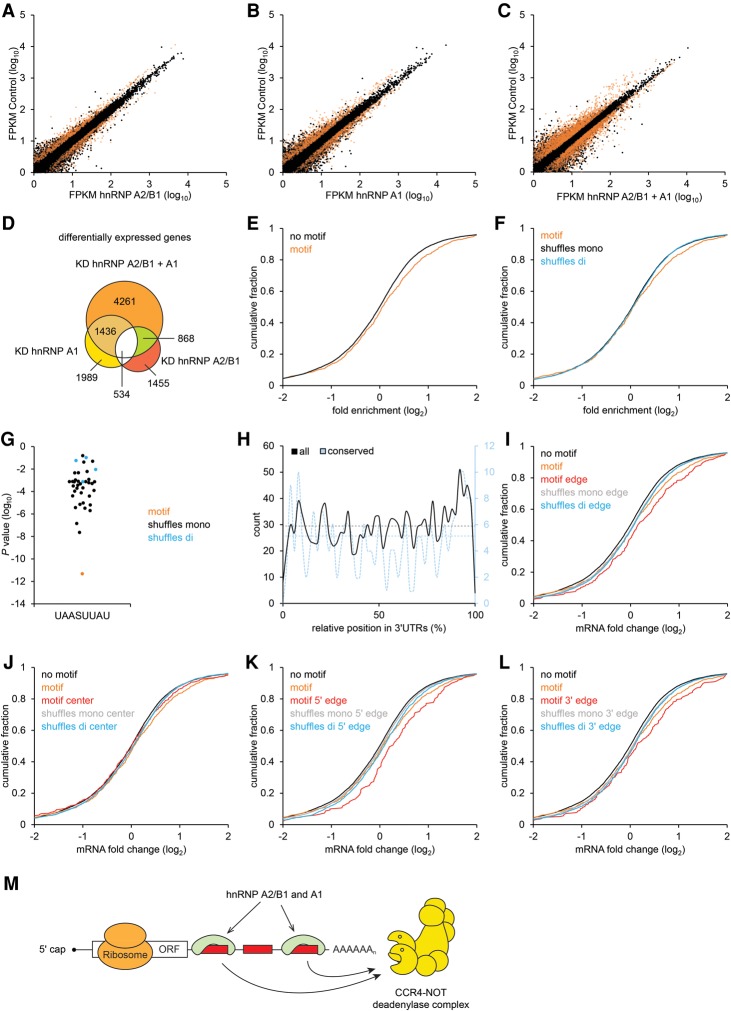
Impact of hnRNPs A2/B1 and A1 on the transcriptome. (*A*–*C*) Expression (average FPKM [fragments per kilobase of transcript per million mapped reads] of replicates) of transcripts detected by RNA-seq from cells depleted (*X*-axis) in hnRNP A2/B1 (*A*), hnRNP A1 (*B*), or both A2/B1+A1 (*C*), each compared with control-treated cells (*Y*-axis), indicating (in orange) transcripts with significant (*P* < 0.05) differences in expression. Transcripts with the motif in their 3′ UTRs were significantly enriched in up-regulated transcripts (*P* < 10^−7^, *P* < 10^−3^, and *P* < 10^−8^, number of cases of up-regulated transcripts with the motif = 69, 77, and 171, for *A*–*C*, respectively, hypergeometric test) and not enriched in down-regulated transcripts. (*D*) Venn diagram illustrating the overlap between differentially expressed transcripts shown in *A*–*C*. (*E*) Cumulative distributions of mRNA fold changes for mRNAs containing the 3′ UTR motif (orange) to all other expressed genes (black) in hnRNP A2/B1+A1-depleted A549 cells. (*P* < 0.001, one-sided Kolmogorov-Smirnov test). (*F*) As in *E* but comparing mRNAs with the motif within their 3′ UTRs with those containing a mononucleotide (black; *P* < 0.05) or dinucleotide shuffled motif sequence (blue; *P* < 0.05). (*G*) Enrichment (*Y*-axis; *P*-values by hypergeometric test) of 3′ UTRs containing the motif (orange dot) in transcripts up-regulated (*Q* < 0.05) in hnRNP A2/B1+A1-depleted cells compared with enrichment of transcripts containing shuffled sequences (mononucleotide and dinucleotide shuffles shown in black and blue, respectively). (*H*) Binned counts of total (black) and conserved (blue) instances of the motif (*left* and *right X*-axes, respectively) with respect to their relative 3′ UTR positions (*Y*-axis). Dashed lines indicate average counts if occurrence were uniformly distributed. (*I*–*L*) Assessment of motif activity at different 3′ UTR positions. Cumulative distributions of changes in mRNA levels, as described in *E*, but restricting analysis to instances of the motif (or control sequences) located within (*I*; *P* < 5 × 10^−5^; *P* < 0.01, compared with shuffles) and without (*J*; not significant) the first and last 300 nt of 3′ UTRs, the first 300 nt (*K*; *P* < 0.001; *P* < 0.05, compared with shuffles,) and the last 300 nt (*L*; *P* < 0.001; *P* < 0.05, compared with shuffles), respectively. (*M*) Model of UAASUUAU-mediated mRNA decay. hnRNPs A2/B1 and A1 bind to UAASUUAU (red rectangles) preferentially at distal sites of 3′ UTRs and recruit the CCR4–NOT complex to mRNAs to promote deadenylation.

To examine global roles of hnRNPs A2/B1 and A1 in mediating motif-dependent mRNA decay, we used RNAi to deplete A2/B1 and A1 singly and in combination, together with control-treated cells, and measured changes in RNA abundances with RNA sequencing (RNA-seq). Replicates of these experiments correlated well (Supplemental Fig. S5). Inhibition of either hnRNP A2/B1 or A1 had a relatively modest impact on the transcriptome; however, inhibition of both proteins resulted in a far more pronounced global change, with many thousands of transcripts present at significantly different levels in the double knockdown (Fig. 7A–D). When considering genes with the motif in their 3′ UTRs, we observed a significant overrepresentation of transcripts containing the motif in genes with increased expression in hnRNP A2/B1+A1-depleted cells, whereas single depletions had only a moderate impact (Fig. 7E; Supplemental Fig. S6). This result was further confirmed by comparing UTRs with the motif with alternative background gene sets comprised of those UTRs with shuffled versions of the motif (Fig. 7F). Indeed, when considering transcripts up-regulated in hnRNP A2/B1+A1-depleted samples, the motif was strikingly enriched compared with each shuffled version (Fig. 7G).

We were interested in detecting any global signatures that might identify the most effective instances of the motif within 3′ UTRs. One possible parameter that we found was the position of the element within the 3′ UTR, with an apparent preference for the element within both terminal regions of 3′ UTRs (Fig. 7H; Supplemental Fig. S7A,B). To examine whether the location of the motif is functionally relevant, we re-examined the RNA-seq data, partitioning transcripts according to the relative location of sequence elements. Strikingly, the locations of effective motifs were significantly enriched at the 3′ and 5′ ends of 3′ UTRs; indeed, elements within the central portion of 3′ UTRs are largely inactive (Fig. 7I–L; Supplemental Fig. S7C–J). To independently assess the possible significance of motif position, we returned to a comparative analysis, which revealed a significant (*P* < 0.05) bias toward preferential conservation of the motif in the 3′ and 5′ ends of 3′ UTRs. Taken together, these results indicate that the motif is most effective when located away from the central region of 3′ UTRs, toward locations where they tend also to be more strongly selectively maintained.

## Discussion

It is well established that post-transcriptional regulatory pathways are of central importance to gene expression; less clear, however, are the identities of regulatory sequences that mediate post-transcriptional regulation and the mechanisms that they direct (Matoulkova et al. 2012). This is in stark contrast to transcriptional regulation, for which comprehensive genome-wide descriptions of regulatory sequences with detailed mechanistic descriptions exist (Mercer and Mattick 2013). Here, we focused on a specific post-transcriptional pathway, identifying the initiating sequence elements, the *trans*-acting factors required for their function, and the downstream factors and events that ensue. This novel pathway is notable in terms of both the number of mRNAs that contain active elements, which number in the hundreds, and the relatively robust regulation that is conferred. In particular, we tested 19 examples individually, all of which were functional; moreover, the degree of repression observed for the 19 examples exceeds that of typical miRNA-binding sites, which are prototypical 3′ UTR-repressive elements of clear biological relevance. Approximately 7% of human 3′ UTRs contain the motif, and we confirmed their widespread efficacy in genome-wide assays; to our knowledge, the motif that we discovered is among the most prevalent 3′ UTR regulatory elements known.

The two sequence elements that we focused on, UAACUUAU and UAAGUUAU, are nearly identical, and we demonstrate that they are functionally interchangeable. These elements promote mRNA destabilization through deadenylation by recruiting the CCR4–NOT complex. While the CCR4–NOT complex also functions cotranscriptionally, regulating RNA polymerase II (Winkler et al. 2006), our data clearly establish that the motif has no effect on transcription. Moreover, the motifs are ineffectual as DNA regulatory sequences and within the nascent transcript. Taken together, our data demonstrate an exclusively post-transcriptional role for the motif, likely functioning in cytoplasmic mRNAs.

The activity mediated by the motif is reminiscent of pumilio-, Nanos-, GW182-, and TTP-mediated regulation in that these proteins and hnRNP A1+A2/B1 recruit the CCR4–NOT complex to mRNAs to promote deadenylation (Fig. 7M; Lykke-Andersen and Wagner 2005; Braun et al. 2011; Van Etten et al. 2012; Bhandari et al. 2014; Chen et al. 2014; Mathys et al. 2014). Indeed, while differential recruitment of the CCR4–NOT complex is likely a major global determinant of mRNA stability (Wahle and Winkler 2013), there is little knowledge regarding how this differential recruitment is accomplished. The motif is AU-rich and thus might be related to AREs, although the motif does not fulfill the established definitions of AREs (Barreau et al. 2005). AREs interact with a variety of *trans* factors, triggering either decreased or increased expression, depending on the specific factors recruited and the identity of the transcript (Barreau et al. 2005). We observed all tested exemplars of the motif to function as repressors, tested in a multitude of 3′ UTR contexts, suggesting that all elements are regulated by the same *trans*-acting factor. Thus, our motifs function rather differently and with more specificity than do AREs; nevertheless, whether they possess a degree of mechanistic overlap with AREs remains to be determined. Future studies will be needed to determine the precise nature of the interactions between hnRNPs A2/B1 and A1 and the CCR4–NOT complex or whether additional proteins are necessary to mediate interactions.

In addition to functional copies of the motif in the *CNOT1* 3′ UTR, other components of major mRNA decay pathways also contain the motif, suggesting that one pathway regulated by the motif is mRNA decay itself. In particular, the motif is found within the 3′ UTRs of the *CNOT4* and *CNOT6* transcripts, which encode additional subunits of the CCR4–NOT complex; within *XRN1*, the major 5′ exonuclease acting on mRNAs; and within *DCP1A*, a component of the decapping complex. Notably, deadenylated transcripts, such as those that result from the action of motif-dependent hnRNP A1+A2/B1 activity, are further degraded by decapping followed by exonucleolytic decay by XRN1 (Garneau et al. 2007). Most importantly, together with *CNOT1*, levels of *CNOT4*, *XRN1*, and *DCP1A* were all elevated in hnRNP A1+A2/B1-depleted cells (Supplemental Fig. S7K). Together, these results are consistent with an elaborate feedback regulatory pathway associating multiple components of canonical mRNA decay pathways and mediated by the motif.

We identified the hnRNPs A2/B1 and A1 as *trans*-acting factors underlying the function of the motif, which belong to the A/B family of ubiquitously expressed hnRNPs (He and Smith 2009). The two proteins are paralogs and share high sequence identity in their two RNA recognitions motifs. Both proteins are primarily localized to the nucleus and possess a wide variety of established roles, functioning in splicing, transcription, and export (Pinol-Roma and Dreyfuss 1992; He and Smith 2009; Huelga et al. 2012). Furthermore, these proteins have been suggested to regulate mRNA translation and stability (Svitkin et al. 1996; Goodarzi et al. 2012). Interestingly and reminiscent of our findings, A2/B1 and A1 have been reported to regulate alternative splicing while also acting in a cooperative manner (Huelga et al. 2012). Both A2/B1 and A1 show broad RNA-binding preferences from G-rich to AU-rich regions (He and Smith 2009) and have been implicated previously as binding within 3′ UTRs (Huelga et al. 2012), although the mechanistic consequences of 3′ UTR binding and the sequence specificity have not been determined prior to this study.

An intriguing aspect of motif-mediated regulation is its position specificity within the 3′ UTR. Active copies of the motif are enriched within the 5′-terminal and 3′-terminal regions of 3′ UTRs, where they tend to be more preferentially conserved than those within the central region. Mammalian 3′ UTRs are notable in terms of both their length, averaging ∼1700 nt, and their propensity to undergo regulated APA, resulting in longer and shorter 3′ UTR isoforms under different conditions. It is well established that such different isoforms confer different post-transcriptional fates to their associated transcripts (Mayr 2015), although it is less well understood how these alternative fates are specified. In general, shorter isoforms tend to result in increased protein levels, a trend only partially explained by loss of repressive miRNA target sites (Mayr and Bartel 2009). We propose that loss of this motif also contributes to increased protein output for shorter 3′ UTR isoforms, as a consequence of increased mRNA stability. Transcripts derived from genes containing distal copies of the motif within their 3′ UTR regions have the potential to elude motif-mediated control when expressed as short isoforms. In contrast, proximal copies of the motif are more likely to function ubiquitously in all 3′ UTR isoforms derived from APA.

It is worth speculating regarding the mechanistic basis for position-specific effects of the motif. Perhaps the preference for active elements toward the 3′ terminus reflects an advantage of proximity to the tail itself, resulting in increased efficiency recruiting the CCR4–NOT complex to the poly(A) tails compared with sites located hundreds or thousands of nucleotides upstream. Such a model, however, cannot also explain the preference for 5′ sites. Interestingly, m^6^A methylation has been reported to occur preferentially toward the 5′ edge of 3′ UTRs, and hnRNP A2/B1 has been shown to bind methylated RNA (Alarcon et al. 2015)—an observation that might explain the 5′ preference for the motif. Finally, perhaps aspects of these position-specific preferences within 3′ UTRs are relatively widespread, as similar preferences influence the efficacy of miRNA-binding sites (Grimson et al. 2007).

In addition to functional testing using a variety of reporter assays, we established that the motif contributes to the control of cytokine expression. Under normal conditions, cytokine genes are transcribed at very low levels (Medzhitov 2008); perhaps the presence of the motif within 3′ UTRs acts as an additional fail-safe, further limiting protein production by increasing the rate of mRNA degradation. Thus, the presence of the motif within cytokine 3′ UTRs may comprise a safeguard mechanism to prevent low-level cytokine production under normal conditions. In contrast, in response to infection, cytokine genes undergo massive transcriptional induction (Medzhitov 2008), a scenario in which regulation by the motif would likely have negligible impact. Thus, the motif would act as a safeguard mechanism to lock down cytokine expression under normal conditions but would not interfere with necessary up-regulation in times of immune challenge. This role of post-transcriptional gene regulation—namely, to help stabilize gene expression levels under “normal” conditions while still allowing rapid elevations in expression when necessary—would allow cells to both maintain equilibrium and respond to environmental or developmental change as needed. Robust and flexible gene regulation, comprised of a suite of both transcriptional and post-transcriptional events, provides additional regulatory logic required for complex eukaryotic cellular function, response, and growth.

## Materials and methods

### Cell culture

A549, HEK293T, Huh7, and MCF7 cells were grown in DMEM (Life Technologies) containing 10% FBS (Sigma-Aldrich) and 1% penicillin/streptomycin (Life Technologies). COS-7/ACC-60 and Ba/F3–IL-12Rβ1–IL-12Rβ2 cells were grown in DMEM with 10% FCS (Life Technologies), 60 mg/L penicillin, and 100 mg/L streptomycin (Genaxxon Bioscience). Ba/F3–IL-12Rβ1–IL-12Rβ2 cells express *gp130* and thus were maintained in the presence of Hyper-IL-6. See the Supplemental Material for a description of the generation of stably integrated cells lines and shRNA-mediated RNAi.

### Plasmids

All constructs were sequence verified; see the Supplemental Material for plasmid descriptions and details of construction.

### Luciferase assays

For each well of a six-well plate, 3.5 × 10^5^ A549 cells were seeded 24 h prior to transfection. Cells were transfected with 10 ng of *pmirGLO* reporter plasmid using Lipofectamine 2000 (Life Technologies) and were harvested 30 h after transfection. RNA transfections used 50 ng each of firefly and Renilla luciferase RNA reporters and were harvested 12 h after transfection. Assays were performed with the dual-luciferase reporter assay kit (Promega) and a Veritas Microplate Luminometer (Turner Biosystems).

### Quantitative RT–PCR (qRT–PCR), 3′RACE, and poly(A) tail length analysis

Total RNA was extracted using TRIzol (Life Technologies) and treated with DNase I (Roche) for 1 h at 37°C prior to phenol/chloroform extraction. For reverse transcription, 0.5 µg of RNA was heated for 5 min to 80°C, and cDNA was generated using RevertAid H Minus reverse transcriptase (Thermo Scientific) and an oligo(dT)_19_ or gene-specific primers for 1 h at 42°C followed by heat inactivation for 10 min at 70°C. qPCRs (for primers, see Supplemental Table S2) were performed with SYBR Green master mix (Life Technologies) using a LightCycler 2.0 (Roche) and incubated for 2 min at 95°C with 35 cycles of 10 sec at 95°C, 25 sec at 60°C, and 25 sec at 72°C. Data were normalized to *GAPDH* or the neomycin resistance gene and analyzed by the 2^−ΔΔCT^ method (Livak and Schmittgen 2001). 3′RACE cDNA was synthesized with a modified oligo(dT) primer [AAGCAGTGGTATCAACGCAGAGTAC(T)_30_VN]; PCR products spanning the 3′ end of the GFP coding sequence and the entire 3′ UTR were generated with gene-specific sense primers and 3′RACE_fw AAGCAGTGGTATCAACGCAGAGT as an antisense primer. Poly(A) tail length was analyzed using the ePAT approach (Janicke et al. 2012) and a fragment analyzer (Advanced Analytical). Briefly, 1 µg of total RNA was incubated with a modified oligo(dT) primer (GCGAGCTCCGCGGCCGCGT_12_) and 5 U of Klenow polymerase (New England Biolabs) for 1 h at 25°C for template extension from the 3′ terminus of the poly(A) tail, followed by reverse transcription using 200 U of SuperScript III (Life Technologies) for 1 h at 55°C. Poly(A) tails were amplified using a gene-specific forward primer and the reverse primer GAGCTCCGCGGCCGCGTT.

### In vitro transcription

*pUC18* firefly luciferase 3′ UTR reporter constructs were linearized with BsaI or SpeI to generate templates for mRNAs with or without a poly(A) tail, respectively; *pUC18* Renilla luciferase reporter constructs were linearized with BamHI. Templates used for the generation of a protected poly(A) tail were generated by PCR. Linearized plasmids were in vitro transcribed with T3 RNA polymerase (Agilent Technologies); capping reactions were performed during in vitro transcription at a 2:1 molar ratio of m^7^GpppG/GTP (Jena Bioscience). *pUC19-S1m* aptamer constructs were linearized with BglII or BamHI to generate RNAs with or without S1m aptamers, respectively, and transcribed with T7 RNA polymerase (Agilent Technologies).

### Western blotting

Antibodies were obtained from Santa Cruz Biotechnology (hnRNP A2/B1, sc-32316; hnRNP A1, sc-32301; and β-actin, sc-47778), Bethyl Laboratories (XRN1, A300-443A; PARN, A303-562A; PAN3, A304-914A; and EXOSC4, A303-774A), Proteintech (CNOT1, 14276-1-AP), Sigma-Aldrich (anti-Flag , F7425; and Vinculin, V9131), and R&D Systems (streptavidin-HRP, DY998; and anti-p40 biotinylated, BAF499). Secondary antibodies were purchased from Thermo Scientific (goat anti-mouse IgG, 31432; and goat anti-rabbit IgG, 31462) and LI-COR Bioscience (IRDye 800CW, 926-32210 and 926-32211). Western blots were performed following the manufacturer's instructions with 15–50 µg of protein and were analyzed with an Odyssey infrared imaging system (LI-COR Biosciences) or ChemoCam imager (INTAS Science Imaging Instruments).

### Cell viability assay

COS-7 cells were transiently transfected with 50–100 ng of IL-12 expression constructs using TurboFect (Thermo Scientific). For each well of a six-well plate, 2.5 × 10^5^ cells were seeded in a total volume of 2 mL of medium for 24 h prior to transfection. Medium was changed 6 h after transfection. Cell culture supernatants of transfected cells were collected 48 h after transfection, filter-sterilized, and stored at −80°C. Cells were harvested and lysed in 50 mM Tris-HCl (pH 7.5), 150 mM NaCl, 2 mM EDTA, 1 mM NaF, 1 mM Na_3_VO_4_, 1% Nonidet P-40, and 1% Triton X-100 supplemented with Complete protease inhibitor cocktail tablets (Roche). Protein concentration of cell lysates was determined by BCA protein assay (Thermo Scientific) according to the manufacturer's instructions.

Ba/F3–gp130–IL-12Rβ1–IL-12Rβ2 cells were washed three times with sterile PBS to remove the cytokine HIL-6. Five-thousand cells were resuspended in DMEM supplemented with 10% FCS, 60 mg/L penicillin, and 100 mg/L streptomycin and cultured for 3 d in a final volume of 100 μL with 5% of conditioned cell culture supernatant of transfected COS-7 cells as indicated. The CellTiter-Blue cell viability assay (Promega) was used to estimate the number of viable cells by recording the fluorescence (excitation, 560 nm; emission, 590 nm) using the Infinite M200 PRO plate reader (Tecan) immediately after adding 20 μL of reagent per well (time point 0) and up to 1 h after incubation under standard cell culture conditions. All of the values were measured in triplicate per experiment. Fluorescence values were normalized by subtraction of time point 0 values and to Ba/F3 cells that express only *gp130*.

### FACS

GFP flow cytometry analysis of ∼10^5^ A549 cells expressing GFP reporter constructs was performed on an BD FACSCalibur cell analyzer (BD Biosciences) and evaluated using FlowJo.

### Polysome profiling

A549 cells (2.5 × 10^7^ cells) were treated with 100 µg/mL cycloheximide (Amresco) for 3 min at 37°C in cell culture medium followed by lysis in 500 µL of ice-cold polysome buffer (10 mM HEPES at pH 7.6, 100 mM KCl, 5 mM MgCl_2_, 5 mM DTT, 100 µg/mL cycloheximide, 1% Triton-X-100). Lysates were centrifuged at 12,000*g* for 10 min at 4°C, retaining the supernatant. For polysome profiling, 15%–45% (w/v) fresh gradient sucrose solutions in polysome buffer were prepared in SW41 ultracentrifuge tubes (Beckman Coulter) using a Gradient Master (BioComp). Five-hundred microliters of the cell supernatant was loaded onto the gradients and centrifuged at 38,000 rpm for 100 min at 4°C in an SW41 rotor. Gradients were fractionated (Isco) at 0.75 mL/min with continual monitoring of OD_254_ values.

### RNA-seq

Total RNA was depleted of ribosomal RNA (rRNA) sequences with the Ribo-Zero Gold H/M/R kit (Illumina). One-hundred nanograms of rRNA-depleted RNA was used to generate RNA libraries using the NEBNext Ultra Directional RNA library preparation kit. Reads were aligned to the genome (hg19) with TopHat 2.0.13 (Trapnell et al. 2009), and differentially expressed genes were identified with CuffDiff 2.2.1 (Trapnell et al. 2013). PRO-seq libraries were prepared as previously described (Kwak et al. 2013). Data were deposited in the Gene Expression Omnibus (GSE76151).

### RNA pull-downs

Cytoplasmic extracts were prepared according to Dignam et al. (1983). Nuclei were precipitated and washed twice with nuclear lysis buffer (NLB; 20 mM HEPES at pH 7.6, 150 mM NaCl, 1.5 mM MgCl_2_, 5% glycerine, 0.5% Nonidet P-40), resuspended in three cell pellet volumes of NLB,lysed by 30 strokes of a dounce homogenizer (B-type pestle), and cleared at 12,000*g* for 10 min at 4°C. For RNA pull-downs, 5 µg of S1m aptamer-containing RNAs was immobilized on Dynabeads MyOne Streptavidin T1 (DSbeads; Life Technologies) for 1 h at 4°C in 500 µL of NLB supplemented with 40 U of RiboLock (Thermo Scientific) and washed four times with NLB, transferring to a fresh tube before the last wash. Four-hundred microgams of lysates were precleared on DSbeads for 1 h at 4°C; pre-cleared lysates were transferred to RNA-loaded DSbeads and incubated for 3 h at 4°C followed by four NLB washes. Proteins were eluted in SDS sample buffer.

### MS

Protein bands from SDS-PAGE gels were cut (∼1 mm^3^) and subjected to in-gel digestion and extraction of tryptic peptides (Yang et al. 2007). For nano-LC-MS/MS with electrospray ionization (nano-LC-ESI-MS/MS) analysis, digests were reconstituted in 20 μL of 0.5% FA and applied to an Orbitrap Elite mass spectrometer (Thermo Scientific) equipped with a CorConneX nano ion source device (CorSolutions LLC) and interfaced with a Dionex UltiMate3000RSLCnano system (Thermo Scientific). See the Supplemental Material for further details.

### Bioinformatics analyses

Eight-nucleotide sequences were analyzed for counts of 3′ UTRs containing each sequence and for 3′ UTRs with conserved instances (defined as presence in aligned human, mouse, rat, and dog sequences). Control sequences for each 8mer consisted of shuffled versions of each sequence, maintaining nucleotide composition and overall abundance in 3′ UTRs or with identical dinucleotide composition. Control sequences were chosen to have similar UGUA composition (the core of the pumilio recognition motif). Control sequences containing a 7-nt match to the seed region of deeply conserved miRNAs (Friedman et al. 2009) were removed. For categorical tests, the list of 3′ UTRs containing the motif was compared against a list of up-regulated transcripts. Enrichment was determined by the hypergeometric test. Briefly, the hypergeometric test determines whether the fraction of all RNA-seq transcripts that are up-regulated is significant, given the fraction of all RNA-seq transcripts that contain the motif. The number of observed intersections between these two fractions is compared against the expected value under the assumption that possession of the motif is independent from up-regulation. For cumulative distribution tests, significance was determined by one-sided Kolmogorov-Smirnov test.

## Supplementary Material

Supplemental Material
