# Supplementary material for: A widespread sequence-specific mRNA decay pathway mediated by hnRNPs A1 and A2/B1
Source: Genes Dev. 2016 May 1;30(9):1070–85. doi: 10.1101/gad.277392.116 (PMC4863738; doi:10.1101/gad.277392.116)
Supplement: Supplemental Material [file supp_30_9_1070__index.html]

Supplemental Material 

# A widespread sequence-specific mRNA decay pathway mediated by hnRNPs A1 and A2/B1

## Supplemental Material

**Files in this Data Supplement:**

- Supplemental\_Material.pdf
- Supplemental\_Table\_S1.xlsx
- Supplemental\_Table\_S2.xlsx
- Supplemental\_Table\_S3.xlsx
- Supplemental\_Table\_S4.xlsx
