# Supplementary material for: A widespread sequence-specific mRNA decay pathway mediated by hnRNPs A1 and A2/B1
Source: Genes Dev. 2016 May 1;30(9):1070–85. doi: 10.1101/gad.277392.116 (PMC4863738; doi:10.1101/gad.277392.116)
Supplement: Supplemental Material [file supp_30.9.1070_Supplemental_Material.pdf]

# Supplemental Material

# A widespread sequence-specific mRNA decay pathway mediated by hnRNP A1 and A2/B1

Rene Geissler[1], Alfred Simkin[1], Doreen Floss[2], Ravi Patel[1,3], Elizabeth A. Fogarty[1], Jürgen Scheller[2] and Andrew Grimson[1]*

[1]Department of Molecular Biology and Genetics, Cornell University, Ithaca, New York 14853, USA

[2]Institute of Biochemistry and Molecular Biology II, Medical Faculty, Heinrich-Heine-University, 40225 Düsseldorf, Germany

[3]Graduate Field of Genetics, Genomics, and Development, Cornell University, Ithaca, New York 14853, USA

*Corresponding Author: Andrew Grimson, 445 Biotechnology Building, Cornell University, Ithaca, New York 14853. agrimson@cornell.edu (607) 254-1307

## Supplemental materials and methods

*Plasmids*

Full-length or 3'UTR fragments of 3'UTRs were PCR-amplified from genomic DNA using Phusion High Fidelity DNA Polymerase (NEB). All 3'UTRs were cloned into a modified version of *pIS1* (Addgene plasmid #12179) through *Xho*I/*Bam*HI sites, located between *Sac*I/*Xba*I in the multiple cloning site. Plasmids containing mutated versions of the elements were generated using the QuikChange Site-Directed Mutagenesis Kit (Agilent Technologies). All mutated sequences are described in Supplemental Table S3. For analyzing luciferase activities, 3'UTRs were cloned from *pIS1* constructs into *pmirGLO* (Promega), inserted between the *Sac*I/*Xba*I sites. To generate *pGFP*-3'UTR plasmids for stably integrated reporter expression, 3'UTRs were excised from pIS1 constructs and inserted between *Xho*I/*Bam*HI of a modified lentiviral *pGFP-Neo* plasmid (Clontech). To generate constructs that contain one copy of a 48 nt fragment derived from either the *SHOX2* or *GPR155* 3'UTRs, oligonucleotides were designed and annealed to one another creating *Xho*I/*Xba*I overhangs, followed by cloning between *Xho*I/*Xba*I sites within a modified version of *pmirGLO*, which contains a T7 promoter and a *Eco*RI site upstream of *Xho*I. Constructs with two copies of the 48 nt fragment were generated with a second pair of oligonucleotides, containing the 48 nt insert flanked by *Xba*I/*Sal*I overhangs, and ligated between *Xba*I/*Sal*I sites into constructs that already contained one copy, resulting in *pmirGLO-SHOX2-48nt-2x* and *pmirGLO-GPR155-48nt-2x*, respectively. To generate constructs with four copies, plasmids containing two copies were digested with *Eco*RI/*Xho*I and *Eco*RI/*Sal*I, respectively. The fragments containing two copies and *Eco*RI/*Sal*I overhangs were inserted between

*Eco*RI/*Xho*I. Constructs with ten copies were generated in the same way, by first cloning a fragment that contains four copies into plasmids already containing four copies, to create eight copies; in a second round of cloning, two copies were inserted to generate constructs with ten copies.

To generate the plasmid *pGFP-Neo-pA-SHOX2*, a polyadenylation signal (*pA*) was inserted downstream of the *GFP* coding sequence and upstream of the *SHOX2* 3'UTR using PCR.

*pIS1*-3'UTR constructs with artificially introduced UAASUUAU motifs (Fig. S1G) were generated by PCR with primers containing the motif.

Plasmids encoding firefly and renilla luciferase mRNAs used for *in vitro* transcription were generated by cloning of a *poly(A)*$_{98}$ fragment between *Eco*RI/*Bam*HI of *pUC18*. Following deletion of the *Eco*RI restriction site, a T3 promoter was inserted together with additional *Eco*RI, *Xho*I and *Bgl*II sites; the firefly luciferase coding sequence was ligated into the *Eco*RI/*Xho*I sites of *pUC18-poly(A)*$_{98}$. 3'UTRs were cloned between *Xho*I/*Bgl*II of *pUC18-FLuc-poly(A)*$_{98}$. A *Bsa*I site was inserted downstream of the *poly(A)*-encoding region, using PCR to generate plasmids for *in vitro* transcription of mRNAs, which ended precisely with a poly(A)$_{98}$ tail. The *renilla* luciferase plasmid was generated by cloning the *renilla* luciferase coding sequence between *Sac*I/*Eco*RI to obtain *pUC18-RLuc-poly(A)*$_{98}$.

*S1m* aptamer-encoding constructs were generated according to Leppek and Stoecklin (2013), using *pUC19* as a vector backbone that contains a newly inserted *Bgl*II site. Four copies of the S1m aptamer were ligated between *Bam*HI/*Bgl*II. *pmirGLO-SHOX2-48nt-4x* was digested with *Sac*I/*Sal*I and the fragment inserted into *pUC19-S1m-*

*aptamer-4x* to generate a T7 promoter-containing construct with four copies of the 48 nt region of *SHOX2* and four copies of the *S1m* aptamer.

The plasmid for expression of *IL-12B* (*pcDNA3.1-FLAG- IL-12B-His*) was described previously (Schröder et al. 2015). Murine *IL-12A* was amplified from I.M.A.G.E. cDNA clone 40126170 (Source BioScience) by inserting *Afl*II/*Not*I sites. The resulting cDNA was cloned via *Afl*II/*Not*I sites into the *pcDNA3.1* expression vector containing an Ig κ-chain leader, an N-terminal FLAG and 6xHis tag at the C-terminus, resulting in *pcDNA3.1-FLAG-IL-12A-His*. The expression vector for Hyper-IL-12 (HIL-12) encodes a fusion protein of murine *IL-12B* followed by a synthetic linker (RGGGGSGGGGSVE) and murine *IL-12A* with an N-terminal FLAG and a C-terminal His tag. The human *IL-12A* 3'UTR was inserted into the *Xho*I/*Bam*HI sites downstream of the *IL-12A* coding sequence to generate the *pcDNA3.1-FLAG-IL-12A-3'UTR* expression construct.

Expression constructs for *CNOT1*, *hnRNPs A2/B1* and *A1* were generated by inserting cDNAs into pcDNA5/TO (Life Technologies) with and without a FLAG-tag coding sequence. For stably integrated expression, the *hnRNP A1* cDNA was cloned into the lentiviral *pGFP-Neo* plasmid (described above) by removing the *GFP* coding sequence. shRNA resistant *pcDNA5/TO-CNOT1* and *pNeo-hnRNP A1* were generated by site-directed mutagenesis of the shRNA target site.

shRNA hairpin plasmids (Supplemental Table S4) were obtained from The RNAi Consortium (TRC) or generated by annealing oligonucleotides to one another, followed by cloning into pTRC005 (TRC) through *Age*II/*Eco*RI.

All plasmids and sequences are available upon request.

*Generation of cell lines with stable expression of shRNAs and GFP constructs*

Cell lines stably expressing shRNAs and *GFP*-3'UTR reporter constructs were generated according to TRC protocols. Viral supernatants were harvested 2 days post transfection of shRNA hairpin plasmids or *GFP* plasmids, and analyzed for viral titers using a Resazurin (Life Technologies) cell viability assay. $4x10^5$ A549 cells were seeded into each well of 6-well plates and infected 24 hours later with virus, using an MOI (multiplicity of infection) of 10. Double infections were performed at an MOI of 10 for each shRNA-expressing virus. Cells were selected in growth media supplemented with puromycin (3 µg/ml) for shRNA constructs or neomycin (1 mg/ml) for *GFP* constructs, respectively. All shRNAs used in this study are described in Supplemental Table S4.

*Rescue of CNOT1 and hnRNP A1 function*

For CNOT1 rescue, Hek293T cells stably expressing *GFP* reporter constructs with ten copies of UAAGUUAU were infected with *CNOT1* shRNA in a 6-well format. 24 h post infection 2 µg of shRNA resistant *pcDNA5/TO-CNOT1* were transfected using TransIT-LT1 (Mirus) and RNA levels analyzed 72 h post transfection. For hnRNP A1 rescue, A549 cells stably expressing shRNA resistant *hnRNP A1* were generated. Following infection with *hnRNP A1* shRNA, rescue effects were analyzed using luciferase assays as described in the materials and methods section.

*Purification of hnRNPs A2/B1 and A1, and in vitro RNA binding.*

Recombinant hnRNPs A2/B1 and A1 were purified as FLAG-tagged fusion proteins using ANTI-FLAG M2 Magnetic Beads (Sigma-Aldrich). Briefly, Hek293T cells (100 mm dish) were transfected with 5 µg of expression constructs at 25% confluence using

TransIT-LT1 (Mirus) followed by lysis in immunoprecipitation buffer (IPB; 20 mM HEPES pH 7.6; 300 mM NaCl; 0.5% NP-40; 5% Glycerol) 48 h post transfection. Lysates were centrifuged for 10 min at 12,000 *g* at 4ºC and cytoplasmic supernatants subjected to FLAG beads for 3 h at 4ºC. FLAG beads were washed twice with IPB followed by two wash steps with IPB supplemented with 1 M NaCl for 10 min at 4ºC. Proteins were eluted with 3X FLAG peptide (Sigma-Aldrich) in 20 mM HEPES pH 7.6 and 150 mM NaCl.

RNA binding of hnRNPs A2/B1 and A1 was performed as described for pull-down experiments with 1.5 µg of S1m-aptamer-containing RNAs in presence of 100-fold excess of tRNAs.

*RNA Cross-Linking and Immunoprecipitation (CLIP) and RNA Immunoprecipitation (RIP) assays*

Hek293T cells stably expressing *GFP* reporter constructs with ten copies of UAAGUUAU were transfected with *pcDNA5/TO-FLAG-hnRNP A2/B1* and *pcDNA5/TO-FLAG-hnRNP A1* expression constructs, respectively, as described above. Cross-linking was performed as described in Jeon and Lee (2011) and proteins were purified according to the purification strategy for the recombinant hnRNPs A2/B1 and A1 without the 1 M NaCl wash step. Eluted proteins were digested with 100 µg Proteinase K (Thermo Scientific) in 20 mM Tris pH 7.5, 150 mM NaCl and 0.5% SDS for 30 min at 37ºC. RNAs were extracted with phenol/chloroform and analyzed by qRT-PCR. RNAs were normalized to an *in vitro* transcribed firefly luciferase mRNA added prior to RNA extraction.

*Mass spectrometry*

Peptide samples (5 μl) were injected into a PepMap C18 trap column-nano Viper (5 μm, 100 μm x 2 cm; Thermo Scientific) at 20 μl/min for on-line desalting, and separated on a PepMap C18 RP nano column (3 μm, 75 μm x 15 cm) installed in the nano device with a 10 μm spray emitter (NewObjective). Orbitrap calibration and nanoLC-MS/MS operation, and dynamic exclusion parameters were as described previously (Yang et al. 2011). Peptides were eluted with a 90 min gradient of 5% to 38% ACN in 0.1% FA at a flow rate of 300 nl/min, followed by a 5 min ramping to 95% ACN/0.1% FA and a 7 min hold at 95% ACN/0.1% FA. The Orbitrap Elite was operated in positive ion mode with nano spray voltage set at 1.6 kV and source temperature at 250°C. The instrument was operated in parallel data-dependent acquisition (DDA) under IT-FT mode using FT mass analyzer for one MS survey scan from m/z 375 to 1800 with a resolution of 120,000 (fwhm at m/z 400) followed by MS/MS scans on top 15 most intensive peaks with multiple charged ions above a threshold ion count of 10,000 in FT mass analyzer. Data were acquired with Xcalibur 2.2 (Thermo Scientific). MS and MS/MS raw spectra were output as MGF files by Proteome Discoverer 1.4 (Thermo Scientific) for database searching (Mascot searching engine 2.5.1, Matrix Science), using the human RefSeq sequence database (33,735 entries, downloaded on 7/25/2007 from NCBInr), and was performed with two-missed trypsin cleavage sites allowed, peptide tolerance of 10 ppm and MS/MS tolerance of 0.6 Da. Variable modifications were: carbamidomethyl modification of cysteine, methionine oxidation, deamidation on asparagines/glutamine residues; filtering parameters were: ≤1% FDR; peptide identity probability of 95%; CI with peptide expect cutoff at 0.05.

*RNA-seq analyses*

For categorical tests, the list of 3'UTRs containing the motif was compared against a list of 'upregulated' transcripts, defined as those whose normalized expression in knockdown conditions was higher than the corresponding expression in control conditions. A hypergeometric test was used to establish whether transcripts containing the motif are more likely to be upregulated than transcripts lacking the motif. For cumulative distribution tests, upregulation and downregulation were defined similarly, but 3'UTRs containing the motif were ranked from those having many fold downregulation to those having many fold upregulation. 3'UTRs lacking the motif were also analyzed in this way, along with 3'UTRs having rearranged versions of the motif as defined above for motif identification. Significance across groups was established by merging groups and comparing summed ranks deriving from one group to those deriving from the other, with the Wilcoxon rank-sum test.

## Supplemental figures and figure legends

Geissler_Fig. S1

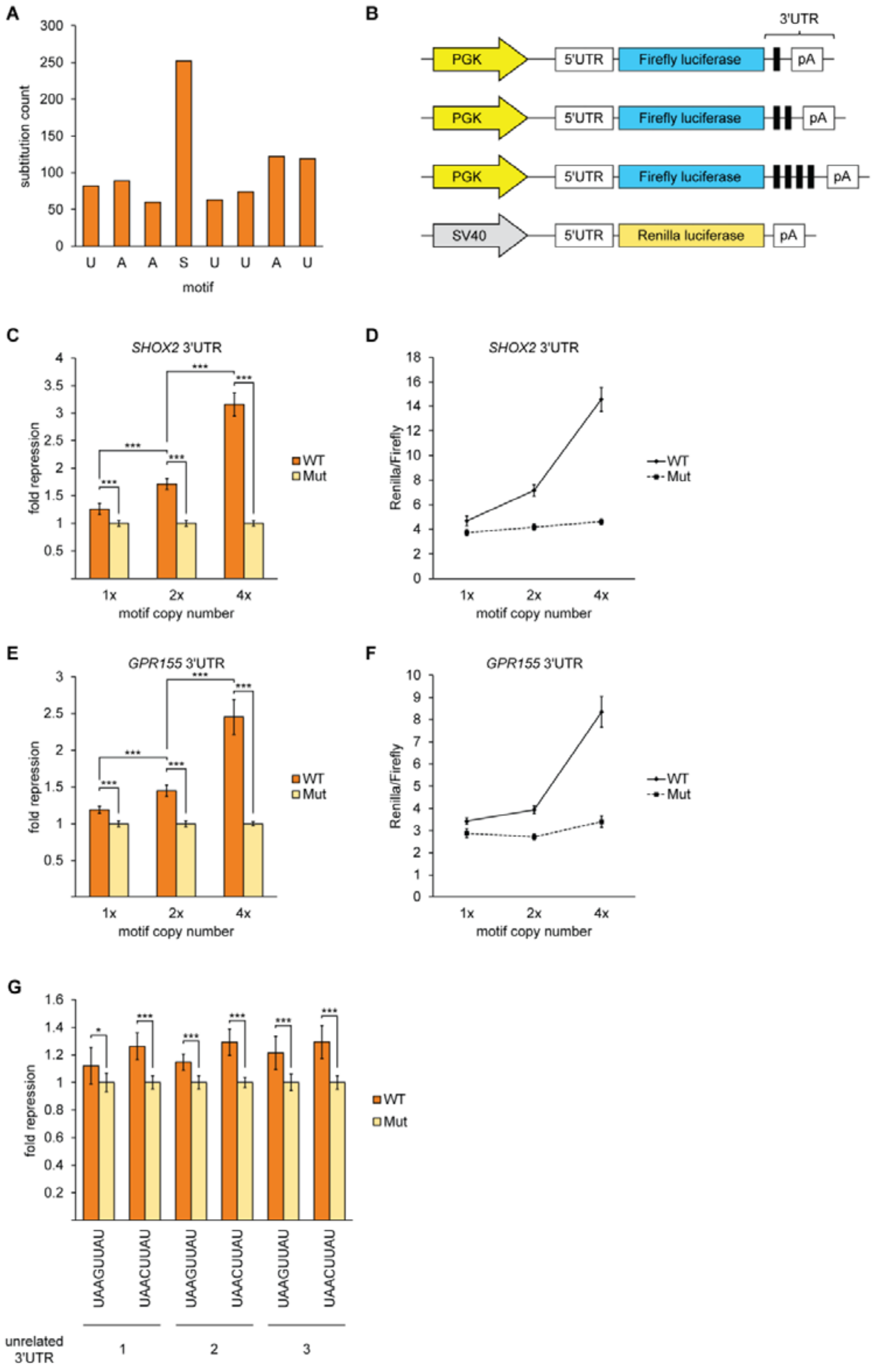

**Figure S1**.

(A) The motif UAASUUAU is most variable at nucleotide four. Ancestral 3'UTR sequences were reconstructed with the DNA Maximum Likelihood program (DNAML; Felsenstein 1989). Ancestrally present instances of the motif were located and nucleotide substitutions inferred along the human, mouse, rat, dog, and human/dog lineages. (B-F) The motif UAASUUAU is repressive, whereas the corresponding mutated control sequence (UAASAAAU) is inert. (B) Schematic presentation of luciferase reporter constructs. Black rectangles indicate locations of UAAGUUAU or UAACUUAU; each element corresponds to a region of 48 nucleotides of the *SHOX2* (UAAGUUAU) or *GPR155* (UAACUUAU) 3'UTRs, with the motif positioned in the center of each 48 nucleotide sequence. (C, E) Luciferase reporter activities of constructs described in (B) that contain the *SHOX2* (C) and *GPR155* (E) 3'UTRs in A549 cells. Luciferase activities of constructs with intact motifs (WT; orange bars) were normalized to otherwise identical constructs with mutated motifs (Mut; yellow). (D, F) Luciferase activities as shown in (C, E) were plotted as ratios of renilla luciferase activities divided by firefly luciferase activities. (G) The sequence UAASUUAU is active in unrelated 3'UTRs that do not contain the motif endogenously. Luciferase reporter activities of 3'UTR constructs with randomly inserted UAASUUAU as described in (C).

Error bars indicate standard deviation (n = 9, Wilcoxon rank-sum test, *** $P < 0.001$, * $P < 0.05$).

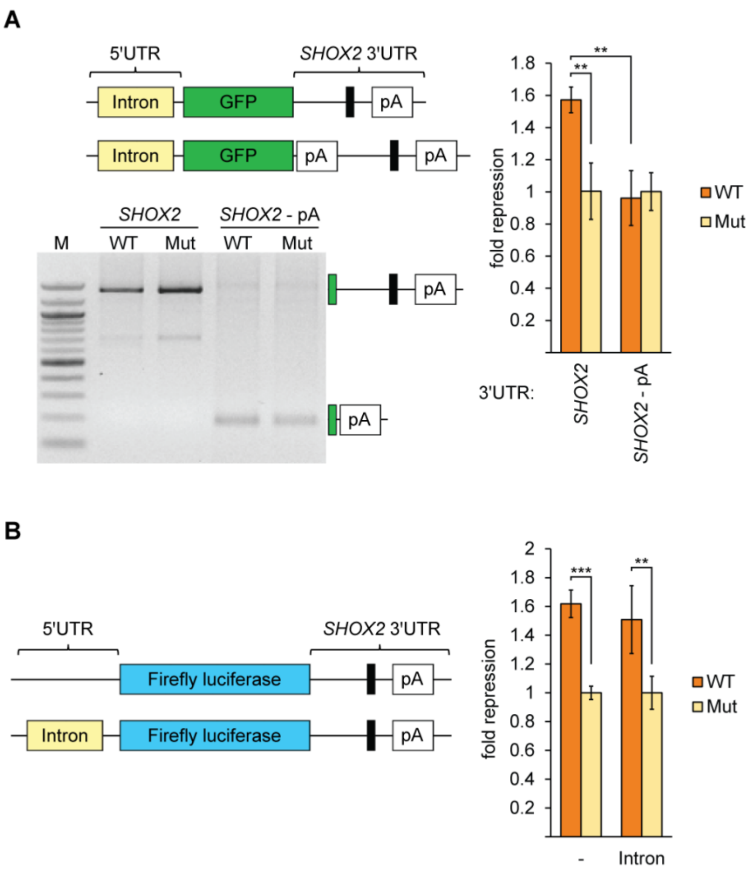


**Figure S2.** Activity of the motif UAASUUAU is restricted to locations within 3'UTRs. (A) Upper left panel - schematic presentation of *GFP-SHOX2* 3'UTR reporter constructs. Black rectangles indicate locations of UAAGUUAU, pA denotes the polyadenylation signal. Lower left panel - 3' RACE of *GFP* constructs described above. Total RNA was isolated and *GFP* mRNAs analyzed by RT-PCR. For 3' RACE, a region spanning the 3' end of the *GFP* coding region and the mRNA's cleavage site was amplified. Fragments were separated on an agarose gel. Right panel - qRT-PCR analysis of *GFP* RNA levels expressed from constructs described above. (n = 3, ** $P$ < 0.01, Student's t-test, error

bars denote standard deviation). (B) The motif UAASUUAU is active in mRNAs that do not undergo splicing. Schematic (left) of luciferase reporters containing, or not containing, an intron, together with reporter data comparing fold repression mediated by the motif in illustrated reporters (right); otherwise as described in Fig. 1C, (n = 9, *** $P < 0.001$, ** $P < 0.01$, Wilcoxon rank-sum test, error bars denote standard deviation).

Geissler_Fig. S3

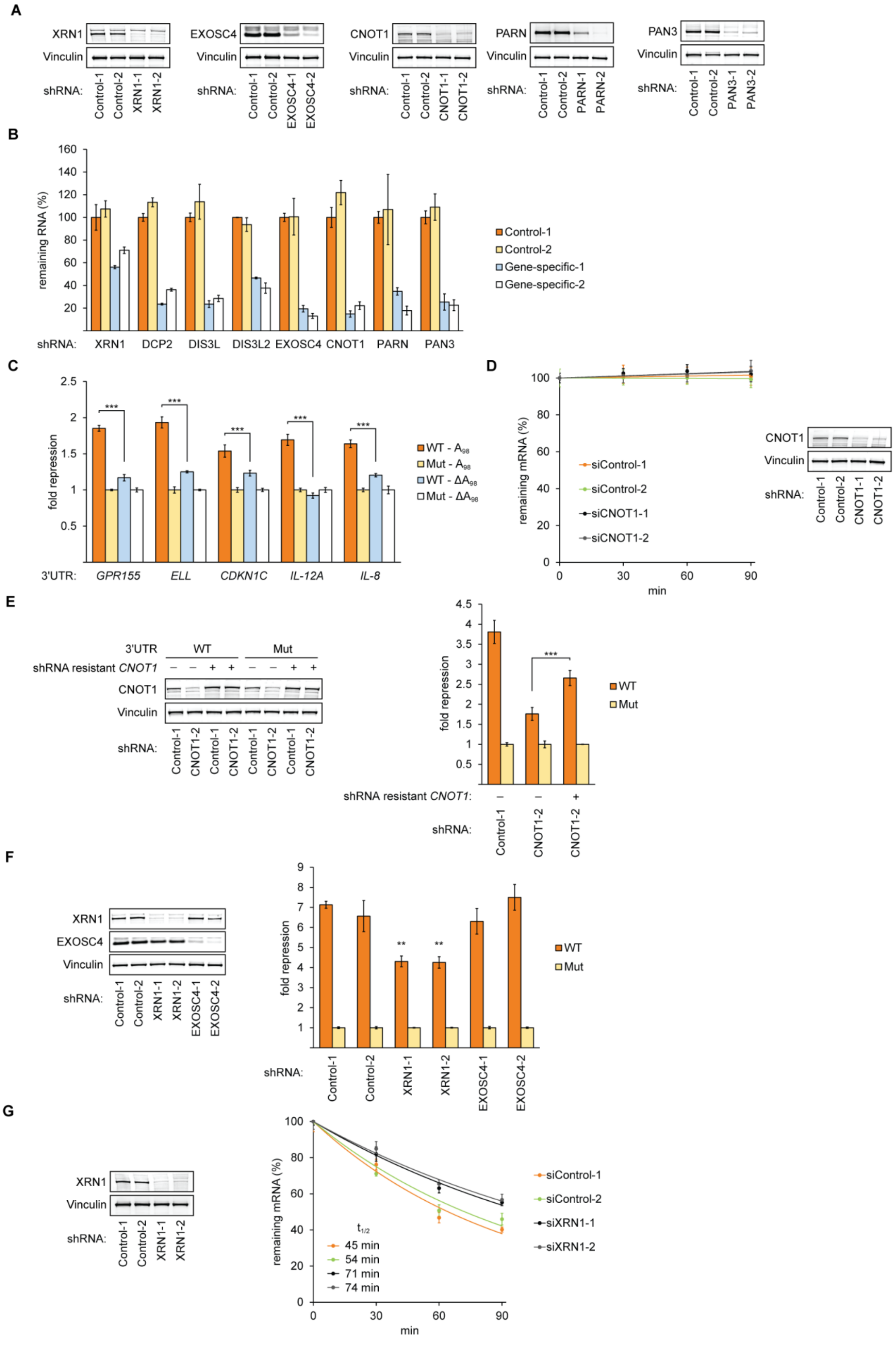

**Figure S3.** The activity of the motif UAASUUAU requires a poly(A) tail. (A) Knockdown efficiency of shRNAs against 5' and 3' mRNA decay factors in A549 cells. Western blots probed with indicated antibodies using XRN1, EXOSC4, CNOT1, PARN and PAN3 depleted extracts; vinculin was probed as a loading control. (B) Knockdown efficiency of shRNAs against 5' and 3' mRNA decay factors in A549 cells. Total RNA was isolated and RNA levels analyzed by qRT-PCR (n = 3, error bars display standard deviation). (C) Firefly luciferase activities of polyadenylated reporter RNAs for 3'UTRs (orange) compared to activities of otherwise identical RNAs containing a mutation disrupting the motif (yellow). Luciferase activities of mRNA constructs with wild-type and mutated motif but without poly(A) tails are shown in blue and white, respectively, (n = 9, *** $P < 0.001$, Wilcoxon rank-sum test, error bars denote standard deviation). (D) Left panel - RNA decay experiment as described in Fig. 5F with *GFP* constructs expressing the mutated motif in CNOT1 and control depleted cells. Right panel - Validation of shRNA knockdown efficacy as described in Fig. 5D. (E) shRNA resistant *CNOT1* rescues the repressive activity of the motif in CNOT1 depleted cells. Left panel - Validation of shRNA knockdown efficacy and overexpression of shRNA resistant *CNOT1* by western blot analysis. Right panel - qRT-PCR analysis of expressed *GFP* RNA levels as presented in Fig. 5C in CNOT1 depleted and shRNA resistant CNOT1 Hek293T cells. (n = 6, *** $P < 0.001$, Student's t-test, error bars display standard deviations).

(F) RNAs with the motif UAASUUAU are degraded via the 5' to 3' decay pathway after deadenylation. Left panel - Validation of shRNA knockdown efficacy of XRN1 and EXOSC4 depletion as described in (A). Right panel - qRT-PCR analysis of *GFP* RNA levels expressed from constructs presented in Fig. 5C in XRN1, EXOSC4 and control

depleted A549 cells. (n = 4, ** $P < 0.01$, Student's t-test, error bars display standard deviations). (G) RNA decay experiment as described in Fig. 5F with *GFP* constructs expressing the wild-type motif in XRN1 and control depleted cells. Left panel - Western blot analysis of XRN1 depleted extracts as described in (A). Right panel - RNA decay analysis of mRNAs analyzed in (E) by qRT-PCR, after treatment with actinomycin D.

Geissler_Fig. S4

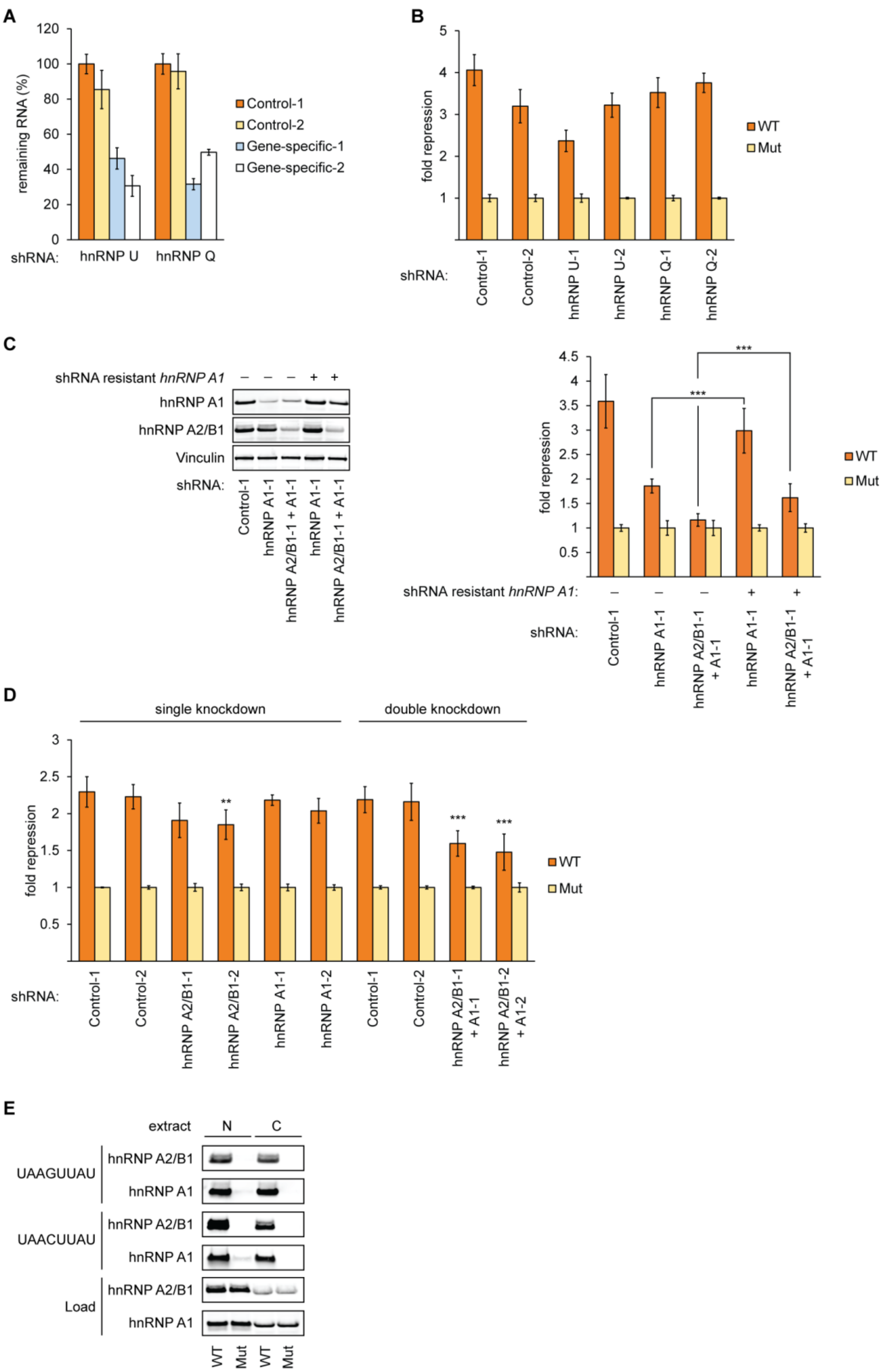

**Figure S4.** Validation of candidate *trans* factors required for repression meditated by the motif UAASUUAU. (A) Knockdown efficiency of shRNAs against hnRNP U and hnRNP Q, performed in A549 cells. Total RNA was isolated and RNA levels analyzed by qRT-PCR (n = 3, error bars represent standard deviation). (B) Luciferase reporter activities of constructs containing 3'UTRs with four copies of the motif, performed in hnRNP U and hnRNP Q depleted A549 cells (n = 9, Wilcoxon rank-sum test, error bars indicate standard deviation). The degree of repression in depleted cells was not significantly different that that observed in shRNA control cells. Additional candidate *trans* factors investigated in equivalent assays included IGF2BP1 and HuR; none of these candidates showed significantly reduced repression for both shRNAs. (C) hnRNP A1 rescue experiment. Left panel - Validation of shRNA knockdown efficacy and overexpression of shRNA resistant *hnRNP A1* by western blot analysis. Right panel - Luciferase assays of reporters containing four copies of the motif in hnRNP A1 depleted A549 cells, as shown in Fig. 6C, and shRNA resistant *hnRNP A1* overexpressing cells (n = 9, *** $P < 0.001$, Wilcoxon rank-sum test, error bars denote standard deviations). shRNA resistant *hnRNP A2/B1* did not rescue the shRNA knockdown, which we attribute to the very low levels of ectopic *hnRNP A2/B1* expression (e.g., see Fig 6F). (D) Luciferase assays of mRNA reporters containing the *SHOX2* 3'UTR as described in Fig. 5B in hnRNP A2/B1 and A1 depleted A549 cells, (n = 9, *** $P < 0.001$, ** $P < 0.01$, Wilcoxon rank-sum test, error bars denote standard deviations). (E) Pull-down experiment, as described in Fig. 6A, with RNAs containing either UAAGUUAU or

UAACUUAU, respectively. Proteins were separated by SDS-PAGE and western blots probed with hnRNP A2/B1 and A1 antibodies.

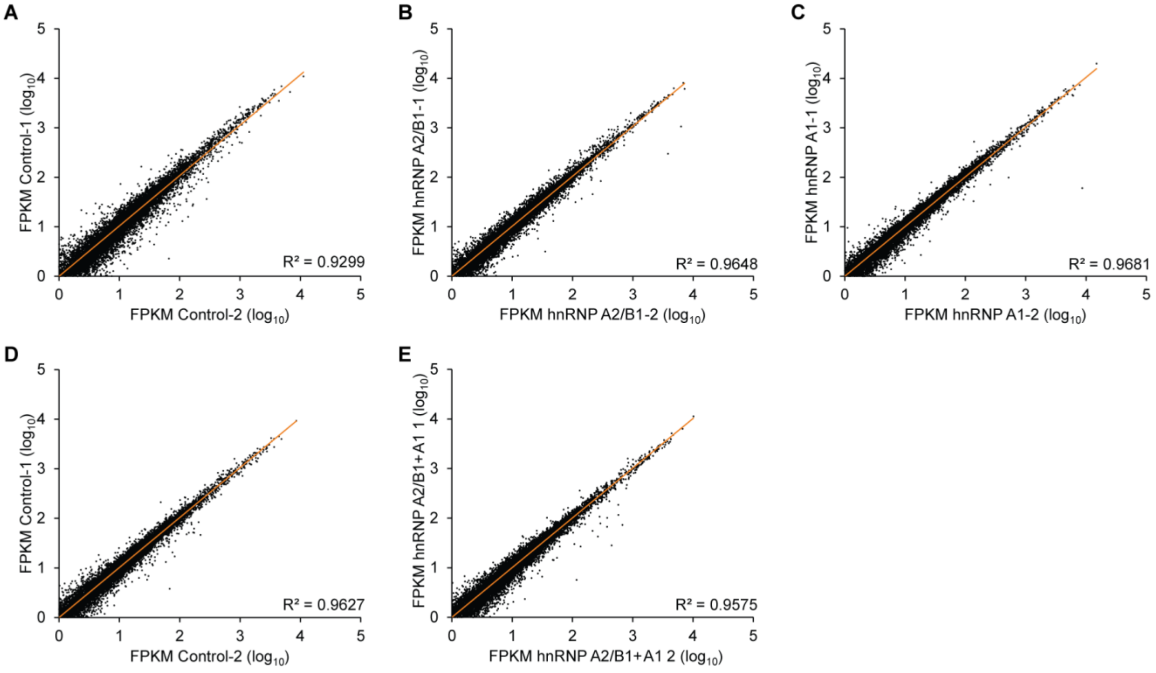


**Figure S5.** Correlation of gene expression profiles in replicates of hnRNP A2/B1 and/or A1 depleted cells. (A-C) Correlation of gene expression from RNA-seq experiments between two biological replicates of control shRNA (A), hnRNP A2/B1 (B) and hnRNP A1 (C) depleted cell lines. All knockdowns were performed with identical MOI (multiplicity of infection); total RNA was isolated and subjected to RNA-seq. The fragments per kilobase of transcript per million mapped reads (FPKM) values of all expressed genes with an FPKM value ≥ 1 are shown; Pearson correlation coefficients are indicated. (D, E). Correlation of gene expression from RNA-seq experiments between two biological replicates of control shRNA (D), and hnRNP A2/B1+A1 depleted cell lines. All knockdowns were performed with identical MOI, which were twice that of the single knockdown experiments.

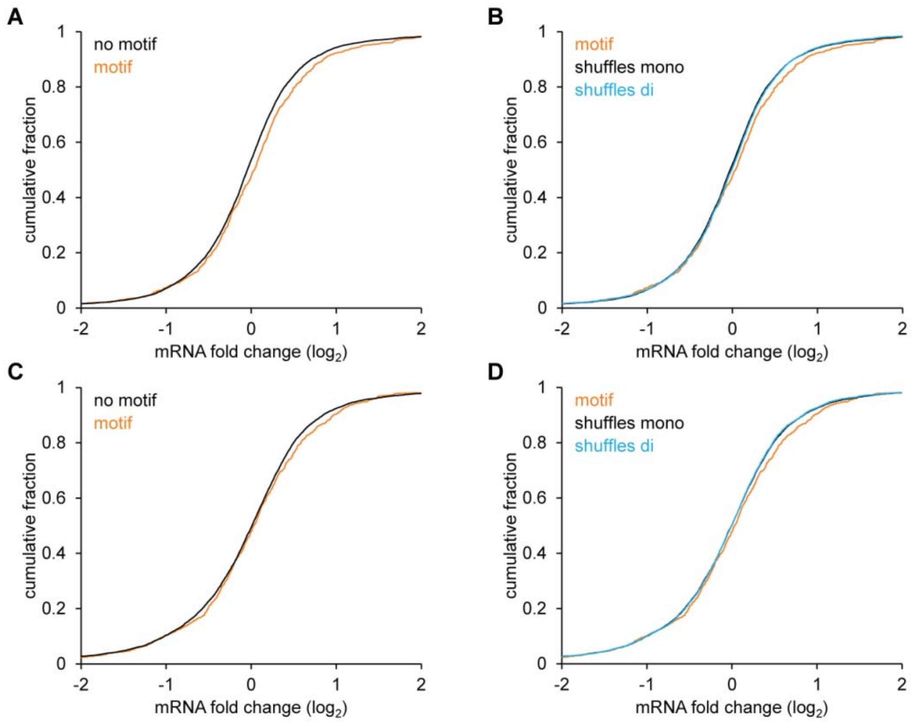


**Figure S6.** Impact of hnRNP A2/B1 and A1 single depletions on the transcriptome. (A, C) Cumulative distributions of mRNA fold changes for mRNAs containing the motif UAASUUAU in their 3'UTRs (orange) and all other expressed genes (black) in hnRNP A2/B1 (A) and hnRNP A1 (C) depleted A549 cells. (B, D) As in (A) and (C), but all mRNAs with the motif were compared to their mono-nucleotide (black) or di-nucleotide shuffles (blue), respectively. Differences between mRNA fold changes were only significant in panel A ($P < 0.05$) and D ($P < 0.05$) (one-sided K-S test).

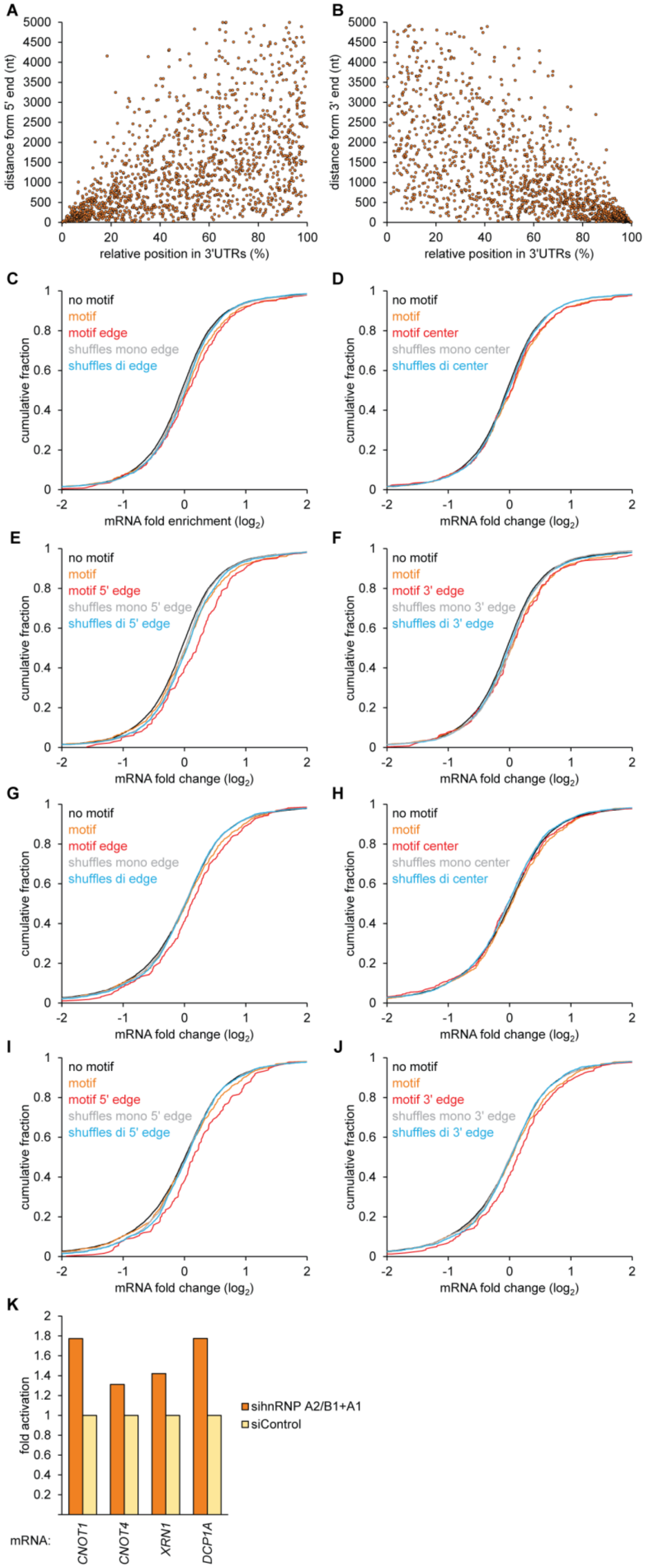
A
distance form 5' end (nt)
relative position in 3'UTRs (%)
B
distance form 3' end (nt)
relative position in 3'UTRs (%)
C
no motif
motif
motif edge
shuffles mono edge
shuffles di edge
cumulative fraction
mRNA fold enrichment (log2)
D
no motif
motif
motif center
shuffles mono center
shuffles di center
cumulative fraction
mRNA fold change (log2)
E
no motif
motif
motif 5' edge
shuffles mono 5' edge
shuffles di 5' edge
cumulative fraction
mRNA fold change (log2)
F
no motif
motif
motif 3' edge
shuffles mono 3' edge
shuffles di 3' edge
cumulative fraction
mRNA fold change (log2)
G
no motif
motif
motif edge
shuffles mono edge
shuffles di edge
cumulative fraction
mRNA fold change (log2)
H
no motif
motif
motif center
shuffles mono center
shuffles di center
cumulative fraction
mRNA fold change (log2)
I
no motif
motif
motif 5' edge
shuffles mono 5' edge
shuffles di 5' edge
cumulative fraction
mRNA fold change (log2)
J
no motif
motif
motif 3' edge
shuffles mono 3' edge
shuffles di 3' edge
cumulative fraction
mRNA fold change (log2)
K
fold activation
sihnRNP A2/B1+A1
siControl
mRNA:
CNOT1
CNOT4
XRN1
DCP1A

**Figure S7.** Impact of hnRNP A2/B1 and A1 single depletions on the transcriptome. (A, B) Relative positions of all instances of the motif UAASUUAU in 3'UTRs (x-axis) were plotted against their distances (y-axis) from the 5' (A) and 3' (B) 3'UTR-termini. (C-J) Activity of the motif UAASUUAU in hnRNP A2/B1 and A1 single depleted cells assessed according to the location of the motif within the 3'UTR; otherwise as described in Fig. S6. (C-F) Motif activity, comparing mRNA fold change between hnRNP A2/B1 and control depleted cells, assessed for 3'UTRs containing the motif in terminal regions (C; defined as first and last 300 nucleotides of a 3'UTR; $P < 0.01$; $P < 0.05$ - compared to shuffles), outside terminal regions (D; $P < 0.05$; not significant compared to shuffles), in 5'-terminal region (E; $P < 10^{-5}$; $P < 0.01$ - compared to shuffles) and 3'-terminal region (F; not significant) (one-sided K-S test). (G-J) As in (C-F), but using hnRNP A1 depleted cells and assessing motif activity in terminal regions (G; $P < 0.01$; $P < 0.05$ - compared to shuffles), outside terminal regions (H; not significant), in 5'-terminal region (I; $P < 0.05$; $P < 0.05$ - compared to shuffles) and 3'-terminal region (J; $P < 0.05$; not significant compared to shuffles). (K) Multiple mRNA decay factors contain the motif in the mRNA's 3'UTR. Expression analysis of mRNAs encoding subunits of the CCR4-NOT (*CNOT1*, *CNOT4*), the decapping complex (*DCP1A*) and the 5' exonuclease *XRN1* in hnRNP A2/B1 + A1 depleted A549 cells based upon RNA-seq ($P < 0.05$ for *CNOT1*, *CNOT4* and *DCP1A*).

## Supplemental references

Felsenstein J. 1989. PHYLIP - Phylogeny Inference Package (Version 3.2). *Cladistics* **5:** 164-166.

Jeon Y, Lee JT. 2011. YY1 tethers Xist RNA to the inactive X nucleation center. *Cell* **146:** 119–133.

Leppek K, Stoecklin G. 2014. An optimized streptavidin-binding RNA aptamer for purification of ribonucleoprotein complexes identifies novel ARE-binding proteins. *Nucleic Acids Res* **42:** e13.

Schröder J, Moll JM, Baran P, Grötzinger J, Scheller J, Floss DM. 2015. Non-canonical interleukin 23 receptor complex assembly: p40 protein recruits interleukin 12 receptor β1 via site II and induces p19/interleukin 23 receptor interaction via site III. *J Biol Chem* **290:** 359-370.

Yang Y, Qiang X, Owsiany K, Zhang S, Thannhauser TW, Li L. 2011. Evaluation of different multidimensional LC-MS/MS pipelines for isobaric tags for relative and absolute quantitation (iTRAQ)-based proteomic analysis of potato tubers in response to cold storage. *J Proteome Res* **10:** 4647-4660.
